# A Comprehensive Review of Sequence and Generative Models in Motor Imagery (MI) Classification for Brain–Computer Interfaces (BCIs)

**DOI:** 10.3390/s26165097

**Published:** 2026-08-11

**Authors:** Muhammad Ahmed Abbasi, Hafza Faiza Abbasi, Muhammad Arsalan, Danish Khan, Andres Annuk, Xiaojun Yu

**Affiliations:** 1 School of Automation, Northwestern Polytechnical University, Xi’an 710072, China; faizaabbasi@mail.nwpu.edu.cn; 2 Department of Electrical Engineering, University of Engineering and Technology (UET), Lahore 54890, Pakistan; m.arsalan@uet.edu.pk; 3 College of Mechatronics and Control Engineering, Shenzhen University, Shenzhen 518060, China; danishkhandirvi@szu.edu.cn; 4 Institute of Forestry and Engineering, Estonian University of Life Sciences, 51006 Tartu, Estonia; andres.annuk@emu.ee

**Keywords:** brain-computer interface (BCI), electroencephalography (EEG), generative adversarial networks (GANs), transformers, motor imagery (MI), variational autoencoders (VAEs), recurrent neural networks (RNNs)

## Abstract

Motor imagery (MI) classification serves as the backbone to brain–computer interfaces (BCIs) by strengthening the communication bridge between the human brain and external peripheral devices. The past two decades have witnessed unprecedented success in MI-BCIs, with applications not only in medical fields but also in several other domains, such as gaming and robotic control. Initially, MI classification primarily relied on classical signal processing techniques that were heavily impacted by signal variations; however, recent trends in deep learning (DL), specifically in sequence-oriented, attention-based, hybrid, and generative architectures such as recurrent neural networks (RNNs), variational autoencoders (VAEs), generative adversarial networks (GANs), and transformers have significantly improved the efficiency and robustness of MI classification. This study presents a comprehensive review of these sequence-oriented, attention-based, hybrid, and generative architectures including RNNs, VAEs, GANs, and transformers, comparing their robustness across various public MI datasets, highlighting their challenges, such as inter-subject variation, low signal-to-noise ratio (SNR), and the obstacles in real-time signal classification. We perform an in-depth analysis on the strengths and limitations of traditional models such as RNNs and LSTMs as well as emergent models such as VAEs and transformers, which have demonstrated superior performance in extracting the intricate patterns of the EEG data with low latency. Moreover, we critically examine the future potential of such models in overcoming current bottlenecks, such as weak generalization on unseen data and high computational load. This study aims to assist researchers in attaining significant insights into the state-of-the-art sequence, attention-based, hybrid, and generative models used in MI classification, thus offering a direction for future innovation.

## 1. Introduction

Communication has been fundamental to the advancement of mankind and the progress of technology throughout human evolution. Human communication has progressed from using basic gestures and vocalizations to complex languages and, more recently, visual media such as video. However, traditional communication modes might sometimes be inaccessible to those with motor disabilities, posing barriers to their daily life activities and social inclusion. In such circumstances, alternative means to communicate hold significant importance. This is where innovative technologies such as brain–computer interfaces (BCIs) emerge, offering novel methods to communicate through neural activity for individuals with physical disabilities [1].

BCIs establish a direct communication link between the human brain and external peripheral devices, circumventing traditional modes of communication [2]. BCIs facilitate effective communication between humans and computers or other external devices by interpreting neural signals to transmit actionable commands. While initially intended to assist individuals with motor disabilities, BCIs have diversified in their applicability, enhancing communication for individuals with motor disabilities and advancing fields such as gaming, neuro rehabilitation, and smart control [3,4].

BCIs are primarily classified into two categories, invasive and non-invasive devices [5], with each possessing distinct applications. To obtain highly accurate neural signals, invasive BCIs need the implantation of electrodes into the brain tissue. Invasive BCIs are ideal for tasks requiring fine motor control, such as operating prosthetic limbs, due to their ability to provide a highly consistent and precise signal. Nonetheless, the significant risks associated with invasive BCIs, including post-operative complications and long-term safety concerns, limit their widespread application in clinical environments. Intracortical neural interfaces (INIs) and electrocorticography (ECoG) are some of the examples of invasive BCIs utilized to assist individuals with severe motor disabilities in regaining their control.

However, non-invasive BCIs, owing to their safety and ease of use, are far more prevalent [6]. Non-invasive BCIs utilize external sensors, particularly electroencephalography (EEG), to monitor brain activity. EEG-based BCIs are favored in both research and practical applications due to their non-invasive nature, enhancing accessibility and utility for a broader range of users. EEG employs electrodes on the subjects’ scalp to non-invasively monitor the neural activity and processes those signals to decode them into actionable commands. However, non-invasive BCIs are certainly less efficient compared to invasive approaches; yet, they remain sufficient for numerous BCI applications, particularly those related to cognitive functions or fundamental motor control [7].

Various paradigms are employed in EEG-based BCIs to analyze cerebral activity. The event-related potential (ERP) is a widely utilized approach that captures brain responses elicited by specific sensory, cognitive, or motor stimuli [8]. ERPs are commonly utilized to design assistive technologies, such as spelling devices that allow users to select letters or words based on their neurological responses to auditory or visual stimuli [9]. The steady-state visually evoked potential (SSVEP) paradigm employs visual stimulation at a specific frequency to facilitate the BCI’s ability to recognize and interpret the user’s attention towards a particular stimulus. SSVEP is extensively utilized due to its robust SNR and capability for rapid transmission speeds [10,11]. Finally, individuals with disabilities exhibit particular interest in the motor imagery (MI) paradigm. Individuals utilizing MI-based BCIs are instructed to envision specific movements, such as hand or foot motion, leading to distinct brain activity patterns. The BCI’s capability to interpret imagined movements and control external devices renders MI a crucial framework for assistive and rehabilitation applications [12]. MI is a particularly promising approach within the various paradigms of EEG-based BCIs, as it offers individuals with motor impairments direct and intuitive control. Unlike ERP and SSVEP, which depend on external stimuli to elicit brain responses, MI engages users in visualizing specific activities without execution, thereby stimulating their cognitive processes [13]. The sensorimotor cortex is activated by this cognitive simulation of motor activity that generates brain signals closely resembling those generated during actual movement. Due to their application in a wide range of conditions, such as spinal cord injuries, stroke, and amyotrophic lateral sclerosis (ALS), MI-based BCIs are one of the most rapidly emerging technologies of the last two decades [14].

Accurate classification of MI signals is of significant importance for the effective functioning of MI-based BCIs. MI classification refers to the process of interpreting distinct patterns in brain while imagining a movement and classifying it into an actionable command [15]. The decoding efficiency of an MI-BCI system depends on the feature extraction and classification techniques used in the framework. Conventional classification methods, such as feed-forward neural network (FFNN) [16], extreme learning machine (ELM) [17], linear discriminant analysis (LDA) [18], and support vector machine (SVM) [19] are commonly utilized in MI-BCIs. Moreover, several advanced models such as convolutional neural networks (CNNs) [20] and long short-term memory (LSTM) [21] networks are also frequently utilized due to their superior capability to capture the intricate patterns of EEG signals. These frameworks are trained repeatedly based on feedback to interpret the neural activity associated with the MI task, thus enabling individuals to operate devices using imagined movements.

However, despite significant growth in the MI-BCI classification industry, numerous challenges in MI classification remain, hindering the widespread adoption of MI-based BCIs. First, the small SNR of EEG data is a primary challenge. EEG signals, acquired non-invasively, are often feeble and susceptible to interference from environmental noise, ocular blinks, or muscular movements [22]. These distortions can obscure the quality of the neural signals, reducing the classifiers’ ability to interpret and differentiate among diverse MI tasks. Moreover, inter-subject heterogeneity in MI signals is also a huge hindrance, since individuals may employ distinct cognitive processes to accomplish identical tasks, leading to incoherent and inconsistent brain patterns. These variations among subjects are a bar to the development of universal MI classification models that require minimal individual calibration and can be used by a diverse array of users. Another significant problem in accurate MI classification is the non-stationary nature of EEG signals. Fluctuations in individuals’ mental states, stress or anxiety levels may induce alterations in brain activity over time, thus reducing the integrity of the acquired signal [23].

Nonetheless, due to the significant potential of MI-BCIs, an increasing volume of research has been undertaken in recent years to reduce the complexity of MI data and enhance the robustness and effectiveness of MI classification [24,25]. The research from the past decade has mainly concentrated on using signal processing and machine learning techniques to decode MI signals. Traditional signal processing techniques, including the continuous wavelet transform (CWT) [26], power spectral density (PSD) [27], Hilbert transform (HT) [28], empirical mode decomposition (EMD) [29], and fast Fourier transform (FFT) [30], have been frequently utilized for feature extraction. These signal processing techniques aim to provide a comprehensive analysis of the neural activity in the brain by converting the complex EEG signals into frequency or time–frequency domains. For instance, FFT transforms the signals from the time domain to the complex frequency domain, making it easier to distinguish several frequency bands, such as mu (8–12 Hz) and beta (13–30 Hz) oscillations, which are commonly associated with MI activity. CWT helps in analyzing the signal over various scales, making it easier to identify fluctuations during MI activity. EMD decomposes the signal into intrinsic mode functions (IMFs) that contain fundamental patterns of the data. Moreover, hybrid combinations of one or more of these signal processing methods have also been experimented with to obtain a more comprehensive analysis of the EEG data [31].

In addition to conventional signal processing methods, some more sophisticated techniques to identify spatial patterns in EEG data have also been developed over the years. Common spatial pattern (CSP) [32] is one of the widely adopted technologies in this domain. The objective of CSP is to optimize the variance disparity between two classes of EEG data, often corresponding to two different MI tasks. CSP has shown considerable performance in enhancing signal quality by decomposing the EEG signal into several spatial components representing the MI task. Zhang et al. [33] utilized CSP in combination with several machine learning methods for MI classification and reported a significant improvement in MI classification accuracy compared to existing methods. Several advanced forms of CSP, such as Filter Bank CSP (FBCSP) [34], have also been introduced to achieve a further improvement in MI decoding performance. The advantage of FBCSP over conventional CSP is that it applies CSP over several frequency bands, thus extracting more discriminative features from the EEG data. FBCSP has shown great success in extracting task-specific features from various frequency bands, thus enhancing MI classification performance [35]. For instance, Cui et al. [36] developed a hybrid framework for MI classification integrating wavelet packet decomposition (WPD) with FBCSP. Their results indicated a higher tolerance to artifacts and variation in the MI signal with a better classification performance. Korhan et al. [37] explored an ensemble learning architecture that uses CSP in combination with various deep learning (DL) technologies to enhance MI decoding accuracy. However, most signal processing methods, including CSP and its variants, are quite sensitive to their own specific parameters and are highly dependent on handcrafted features. In addition, traditional machine learning methods, while effective to some extent, are not efficient enough to handle the complexity and non-stationary nature of EEG data very well [38].

**Figure 1:**
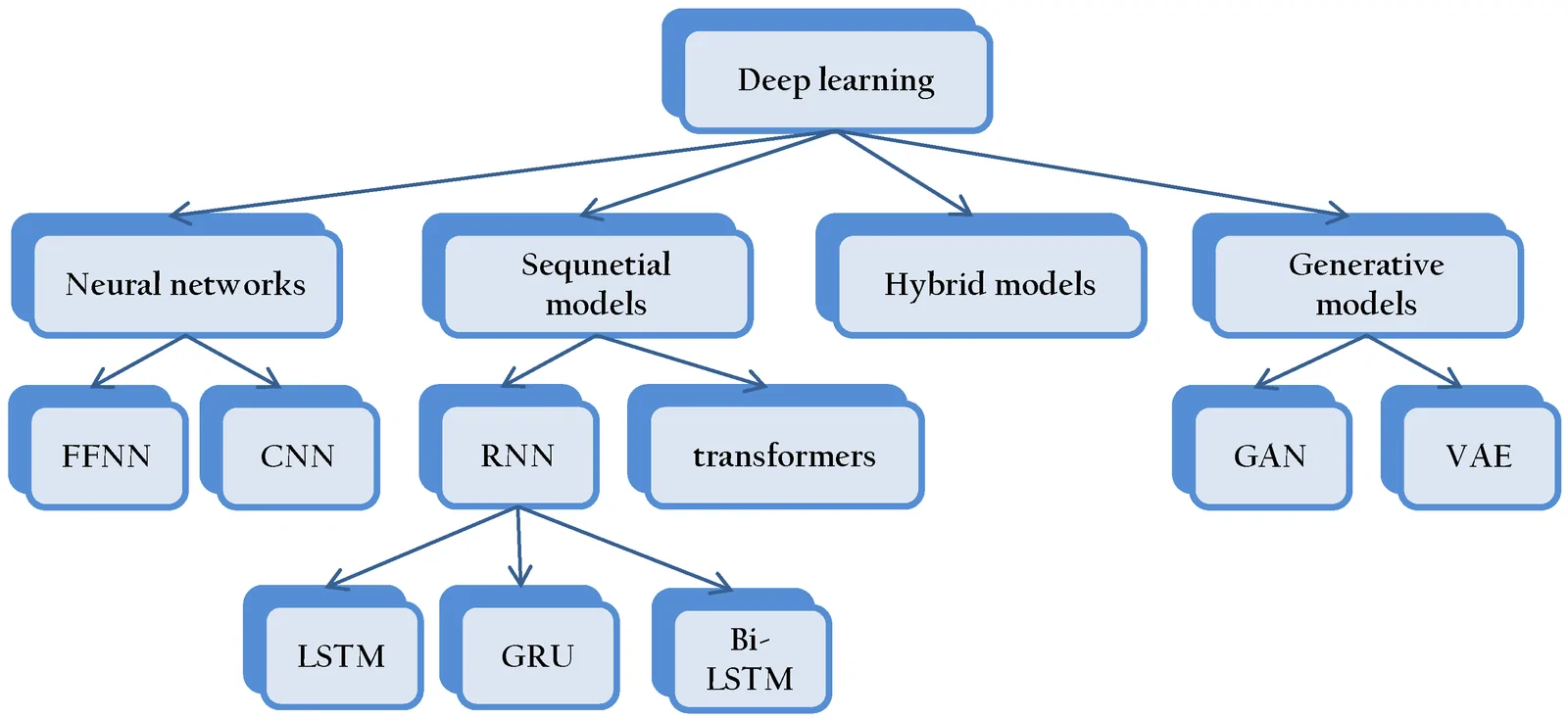
Classification of deep learning techniques.

In the past few years, DL methods have been continuously explored in the MI classification domain due to their autonomous nature, extracting the intricate patterns of data without the need for extensive feature engineering or preprocessing. The classification of deep learning techniques is shown in Figure 1. Numerous DL-based architectures have been designed and implemented for MI classification in the last decade [31,39]. The results of the research studies have shown exceptional performance, particularly in accurately representing the intricate temporal and spatial dynamics of EEG signals. Among DL-based techniques, CNNs are the most frequently utilized networks. CNNs are DL-based architectures that are designed to mimic the human brain and extract the patterns in a way the human brain processes information. CNNs are widely used in MI classification due to their proven success in object detection, computer vision, and other intelligent applications. Schirrmeister et al. [40] introduced a deep and a shallow CNN architecture to extract the complex dependencies in EEG data. Similarly, Lawhern et al. [41] introduced the state-of-the-art EEGNet model, a streamlined CNN consisting of separable and depth-wise convolution layers to extract EEG patterns and at the same time reduce complexity by using separable convolution layers.

In addition to CNNs, sequence models such as gated recurrent units (GRUs) and long short-term memory (LSTM) networks have also shown promising results for MI categorization. Since EEG data is inherently sequential, these models are adept at capturing the temporal dependencies inherent in time-series data. Khademi et al. [42] attained high accuracy in classifying MI tasks by modeling the spatiotemporal correlations in EEG data using LSTM networks. Li et al. [43] enhanced MI classification performance by developing hybrid architectures that integrate LSTMs and CNNs to simultaneously capture spatial and temporal data. Moreover, transformers were recently introduced for natural language processing (NLP); and owing to their huge success in NLP as well as in other domains, such as Vision Transformer (ViT) in computer vision, they have lately been applied to the MI classification domain as well. Transformers employ self-attention mechanisms to more efficiently extract long-range dependencies in EEG data, unlike RNNs and LSTMs, which lose the sequential information over time [44]. Wang et al.’s [45] transformer-based model outperformed traditional RNNs and LSTMs in classifying EEG signals, particularly in multi-class datasets. Luo et al. [46] proposed the shallowest transformer capable of achieving high subject-independent classification performance and also introduced a novel EEG mirroring technique to augment the EEG data. Moreover, Song et al. [47] have also proposed a transformer-based architecture to extract both spatial and temporal features from EEG data and have quoted exceptional performance.

Other than sequence models, generative models such as variational autoencoders (VAEs) [48] and generative adversarial networks (GANs) [49] have also shown promise in addressing certain inherent challenges related to MI classification, including variability and data scarcity. Introduced by Goodfellow et al., GANs consist of a discriminator network and a generator network that are trained in opposition to each other. GANs have been employed to generate synthetic EEG data for MI classification, augmenting the training dataset and improving the robustness of MI classifiers. Roy et al. [50] examined the application of GANs to generating authentic EEG signals and revealed that incorporating synthetic EEG data into training datasets enhances MI classification performance, especially in scenarios with limited data availability. Moreover, VAEs have also been explored for MI categorization, specifically for learning latent representations of EEG data. VAEs can improve classification models by encapsulating the fundamental structure of EEG data within a lower-dimensional compressed representation. Lee et al. [51] significantly improved the efficacy of MI classification models by employing a VAE-based framework to extract pertinent characteristics from raw EEG data. VAEs can enhance classification accuracy by reducing the impact of noise and variability in EEG data via the extraction of robust latent representations.

The integration of generative and sequence models into MI-based BCIs has bought a significant breakthrough in the MI classification field. These models not only aim to enhance classification performance but also address significant challenges that have historically hindered MI classification, including data scarcity, signal unpredictability, and the necessity for extensive training. These models are likely to gain more significance as research advances in developing MI-based BCIs that are more reliable and user-friendly for diverse users. The rapid advancements in these models have significantly advanced the interpretation of MI signals; nonetheless, this high volume of research necessitates a thorough evaluation of their effectiveness. This work systematically examines and assesses recent research employing sequence-oriented, attention-based, hybrid, and generative models for MI classification in terms of accuracy and computational complexity. Consequently, the four principal questions arising from the analysis of the examined literature constitute the focus of this review:

- Which models produced the best MI classification results? Our aim is to identify which architectures have consistently outperformed others by analyzing the performance of various architectures across multiple studies.
- To what degree have sequence-oriented, attention-based, hybrid, and generative models progressed over time in achieving subject-independent classification? Given that subject-independent classification is crucial for the advancement of scalable and generalized MI-BCIs, it is imperative to analyze how these models have tackled the challenges presented by inter-subject variability.
- To what extent and in which capacity have generative models contributed in MI classification? Our aim is to identify how generative models can be used to enhance the MI classification performance.
- Is there a definitive correlation between a model’s performance and its complexity? It is essential to determine whether the increasing complexity of DL models necessarily yields better classification results or if compact architectures would also suffice in some contexts.

This review is organized as follows: Section 2 mentions the resources and methodologies employed to conduct this review. This includes the selection criteria for the relevant publications, the datasets employed in the research, and the evaluation methodologies implemented. Section 3 provides an overview of the MI datasets, including their acquisition process, the challenges associated with their recording, and the key details of some of the common MI-BCI datasets used in the publications discussed in this study. Section 4 provides an overview of a standard MI classification framework, including its validation and significance testing. In Section 5, we briefly discuss the working phenomenon of several sequence, attention-based, hybrid, and generative models, including RNNs, VAEs, GANs, and transformers. Moreover, it also includes a discussion of their fundamental principles, their applications in MI decoding, and their shortcomings. Section 6 includes an analysis of the selected studies used in this review: a comparative analysis and performance evaluation. Moreover, the four review questions of this study are also discussed in this section. This section also discusses some of the critical challenges in MI-BCIs and their potential solutions. Furthermore, applications of MI-BCIs in medical and non-medical domains are also a part of this section. Finally, Section 7 provides the limitations of this review and a brief conclusion highlighting the key findings.

## 2. Materials and Analysis Methods

### 2.1. Selected Research Articles

**Figure 2:**
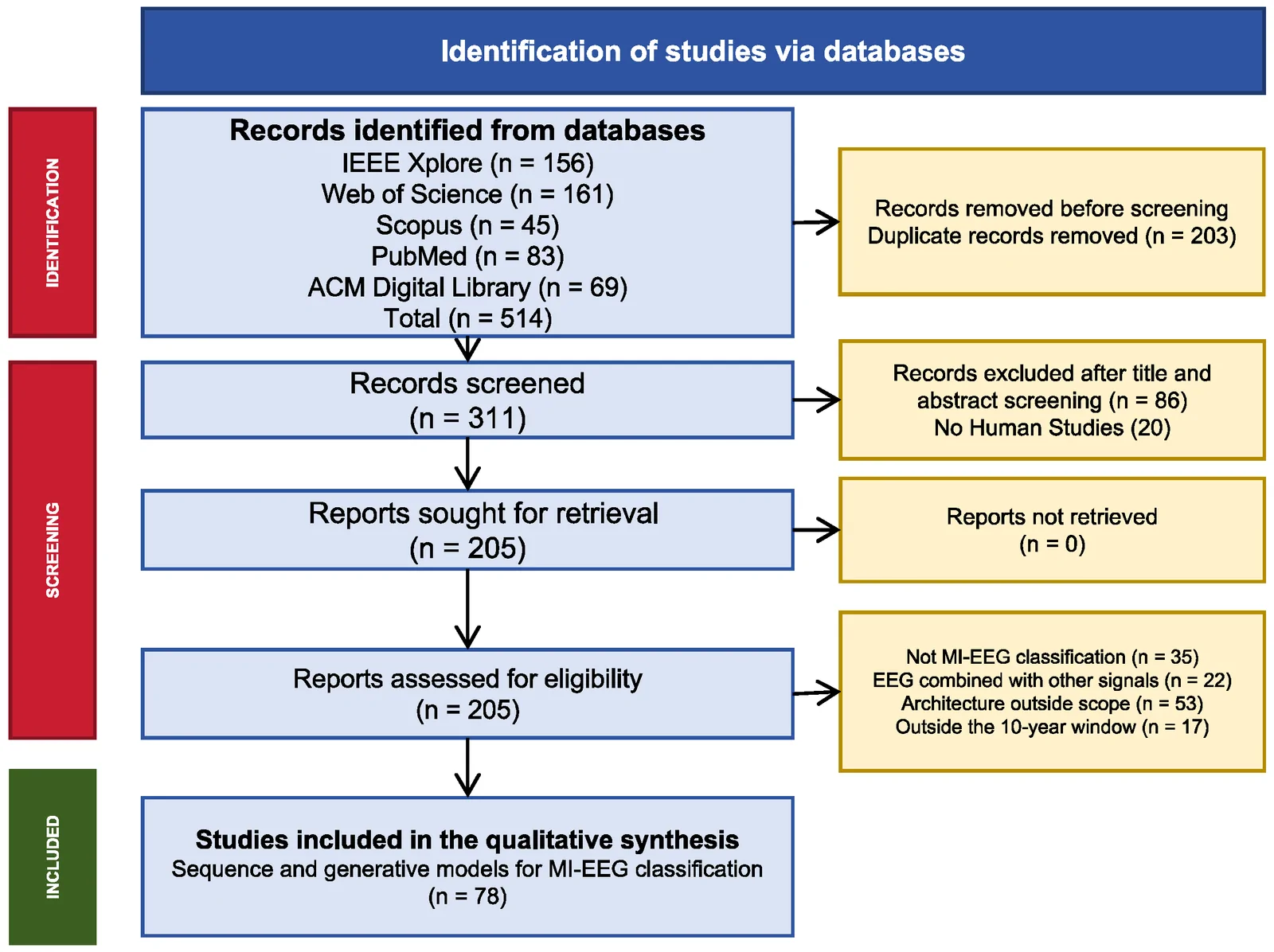
PRISMA 2020 flow diagram showing identification, filtering, and selection of relevant studies.

**Figure 3:**
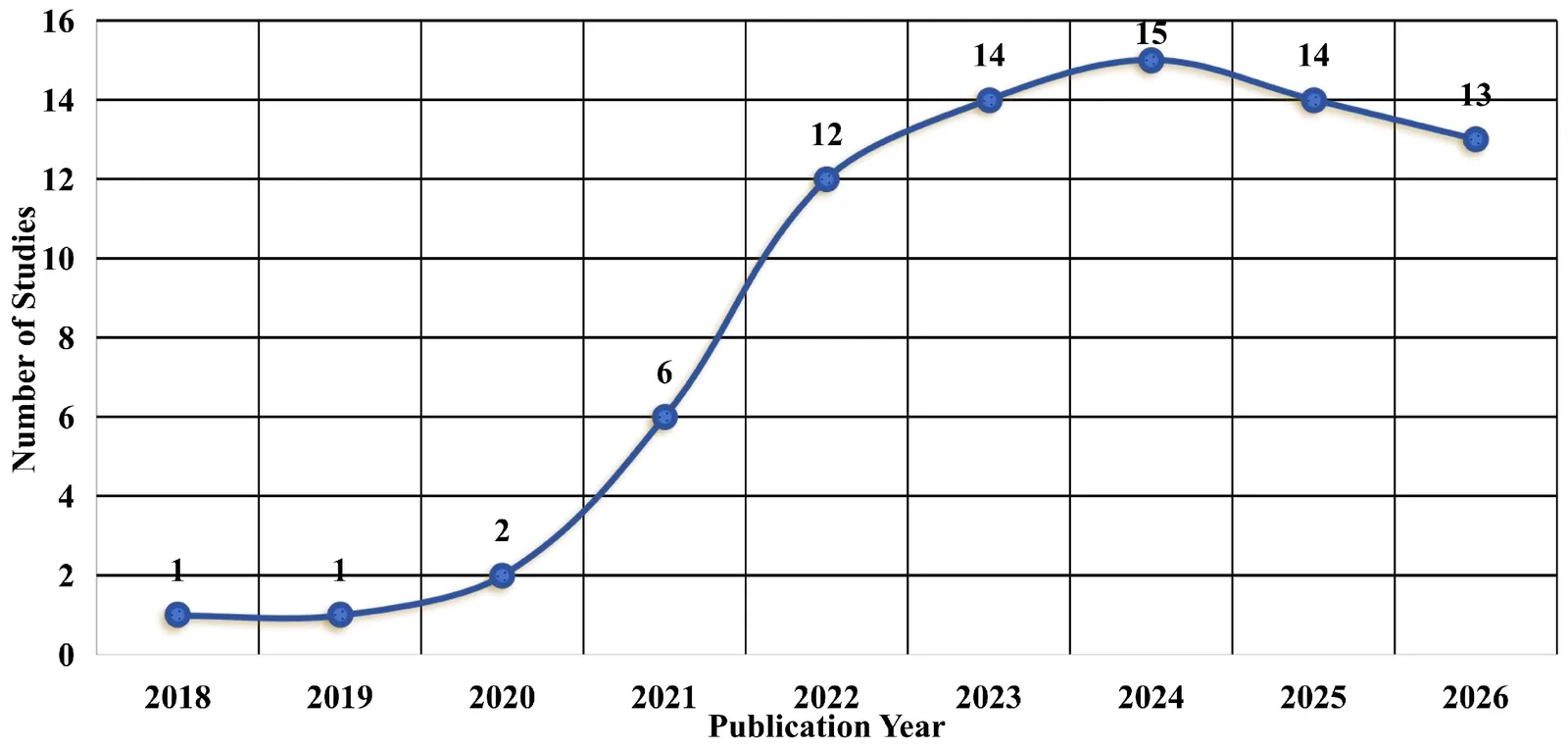
Number of included studies using sequence-oriented, attention-based, hybrid, and generative models over the years.

**Figure 4:**
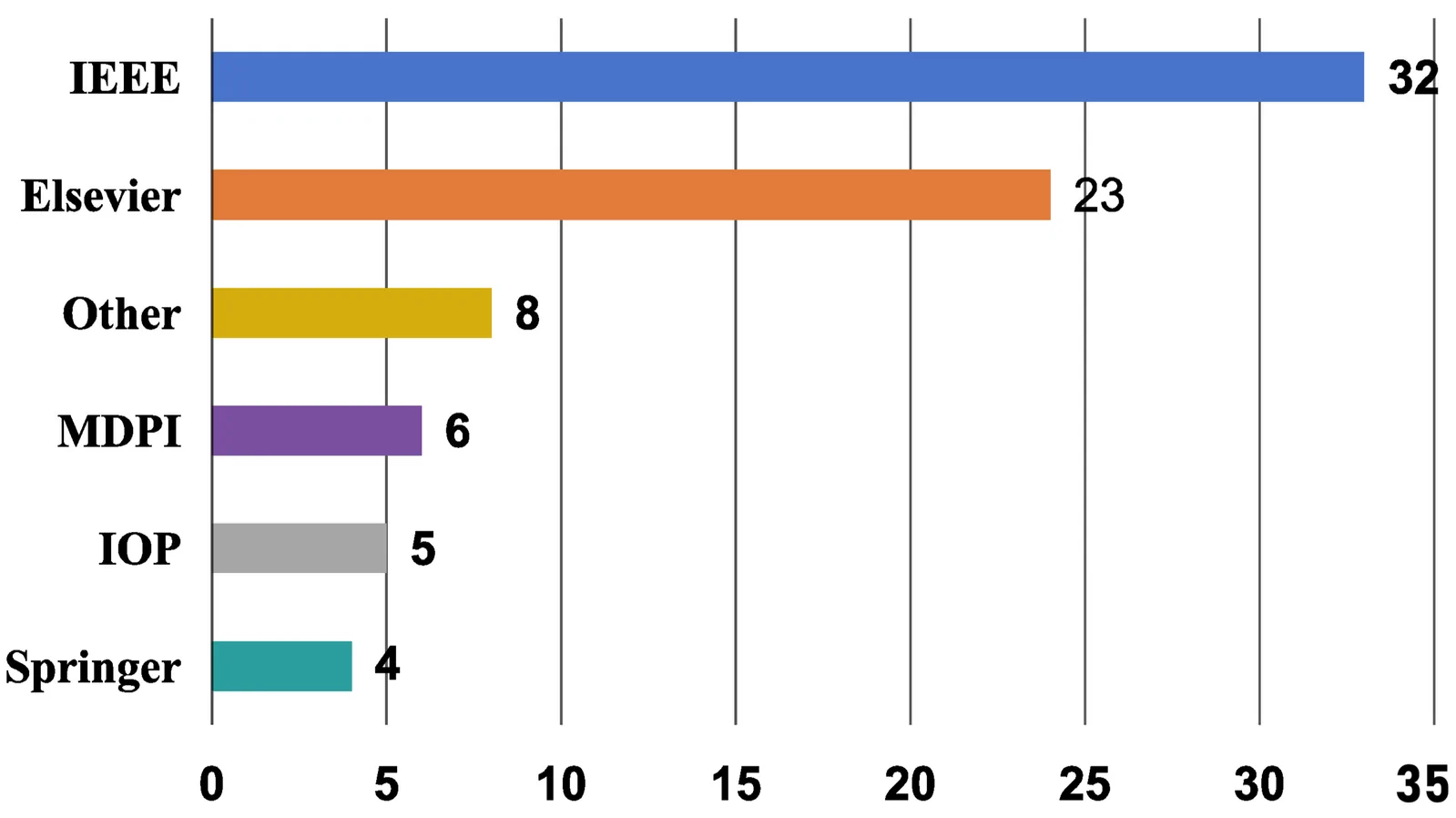
Publishers of the selected studies.

This study was conducted as a systematic review with narrative synthesis. The search method, screening process, eligibility assessment, and the reporting procedures followed the PRISMA 2020 [52] reporting guidelines. Our search strategy, as shown in Figure 2, essentially contains three key steps:

1. In the first step, we searched for relevant studies on several popular research databases, including PubMed, Web of Science, IEEE Xplore, and ACM Digital Library. The relevant articles were searched under the following keywords:
   (“Motor Imagery” or “Motor Imagination” or “Imagined Movement” or
   “MI”) and
   (“EEG” or “Electroencephalography”) and
   (“Recurrent Neural Network” or “RNN” or “LSTM” or “GRU” or
   “Transformer” or “Self-Attention” or “Attention Mechanism” or
   “Sequence-to-Sequence” or “Encoder–Decoder” or “Generative
   Adversarial Network” or “GAN” or “Variational Autoencoder” or “VAE”
   or “Autoencoder”) and
   (“BCI Competition IV 2a” or “BCI Competition IV 2b” or “OpenBMI” or
   “PhysioNet” or “High Gamma Dataset” or “HGD”)
  Our search resulted in 156 articles from IEEE Xplore, 161 articles from WoS, 45 from Scopus, 83 from PubMed, and 69 from ACM digital Library.
2. After obtaining 514 articles from the research databases using the relevant keywords, the duplicates were removed, reducing the number of studies to 311.
3. In the final step, we further narrowed down our scope of analysis by excluding studies that did not fit the following criteria:
  - Electroencephalography:
    Pure EEG signal without any combination with other signals (e.g., those containing electroculography signals).
  - MI: Only MI signals.
  - Sequence-oriented, attention-based, hybrid, or generative models: Architectures including RNNs, GRUs, LSTMs, temporal CNN-RNN hybrids, transformers, self-attention architectures, encoder–decoder models, VAEs, GANs, autoencoders, and related hybrid architectures.
  - Time: Studies from the past ten years only.
  Based on these criteria, studies containing signals from other sources, such as ECoG, or involving tasks other than MI, such as emotion recognition, were removed. Furthermore, studies published more than ten years ago were also omitted. This filter reduced the number of studies to 78 only. A graphical illustration of the number of studies published over the years can be seen in Figure 3, where it can be seen that a high volume of research has been carried out in the past few years for MI classification using sequence and generative models. Figure 4 indicates the publishers of the selected studies.

### 2.2. Retrieved Data

We retrieved the following data from our selected articles:

1.   Preprocessing strategy: The preprocessing technique used before applying any feature extraction or classification model. Common techniques include band-pass filtering, normalization, augmentation, and noise removal using MSPCA, ICA, CAR, etc.
2.   Proposed architecture: The core architecture proposed in the study.
3.   Testing setup: Whether the proposed model was tested in a subject-specific, cross-subject, or subject-independent setup.
4.   Performance: The performance of the model in terms of accuracy, kappa value, or other related metrics.
5.   Journal category: The publisher of the paper, such as Elsevier, IEEE, Springer Wiley, MDPI, IOP, Hindawi, or Frontiers.
6.   Model type: The base model behind the architecture, such as VAEs, GANs, RNNs, LSTMs, CNNs, LSTMs, transformers, or hybrid architectures.
7.   Classifier: The classifier used for feature classification into MI tasks, such as FFNN, SVM, ELM, etc.
8.   Publishing year: The year in which the article was published.
9.   Input data type: The form in which data was input to the designed model, such as raw data, spectral images, or topological maps.
10. Computational cost: The number of parameters in the model.
11. Training time: The time required to train the model.
12. Datasets: The MI datasets used to validate the model. The following information about the dataset was extracted:
  - The frequency of the acquired signal.
  - The timing window of the MI signal.
  - The number of subjects.
  - The tasks performed (such as left hand/right hand).

## 3. MI-BCI Database

### 3.1. MI Dataset Acquisition and Challenges

MI dataset acquisition involves recording the brain signals of an individual generated during imagination of a movement. During this process, non-invasive techniques such as EEG are used to study brain activity during imagination of these movements. This process involves putting electrodes on the subjects’ scalp to record the electrical potentials representing the motor activity. The effectiveness of the collected data depends on several factors, including the MI task at hand, the BCI literacy of the subject, and the specific experimental protocols followed during the recording.

MI data is often hindered by several internal and external artifacts that degrade the quality and reliability of the acquired signals. These artifacts include artifacts from muscular activity, ocular movements, or heart rhythms that may interfere with the obtained MI signal, thus resulting in contaminated data. In addition, environmental factors such as interference from surrounding electronic devices might also affect the quality of MI data. The inherent MI signal differences among individuals and the positioning of electrodes may introduce additional complexities, thus compromising the data integrity. Moreover, as EEG is non-invasive, the SNR in MI data might be reduced significantly, leading to certain artifacts dominating the EEG signal. Thus, preprocessing techniques are employed to reduce the impact of artifacts and enhance the quality of MI data.

### 3.2. MI Dataset Preprocessing

As mentioned earlier, MI data is contaminated by several artifacts that reduce the integrity of the data. Thus, preprocessing is of significant importance to improve the reliability of the data before analysis.

Band-pass filtering [53] is one of the most common techniques used to isolate the signals of the desired frequency range from the acquired signal. As mentioned by several studies in the literature [54,55,56], alpha (8–12 Hz) and beta (13–30 Hz) rhythms are more commonly associated with MI tasks. So, a band-pass filter is usually employed to extract the signal within this frequency range. Independent component analysis (ICA) and common average reference (CAR) are some of the frequently utilized preprocessing strategies for filtering the EEG signals of interest. Ref. [57] provides an in-depth analysis of several preprocessing techniques to remove artifacts from EEG data.

### 3.3. Common MI-BCI Datasets

Several public MI datasets have been recorded to date; they are paving the way for research and development in BCI and neuroscience. These datasets are a means to provide standardized resources that are easily accessible for testing and validating machine learning and DL models for MI classification. They are collected in a controlled environment with uniform recording conditions for all subjects. The data is recorded by placing electrodes on the subject’s scalp, and a specific MI task is performed by the subject. The corresponding signal is recorded and can be used for designing MI classification algorithms.

**Figure 5:**
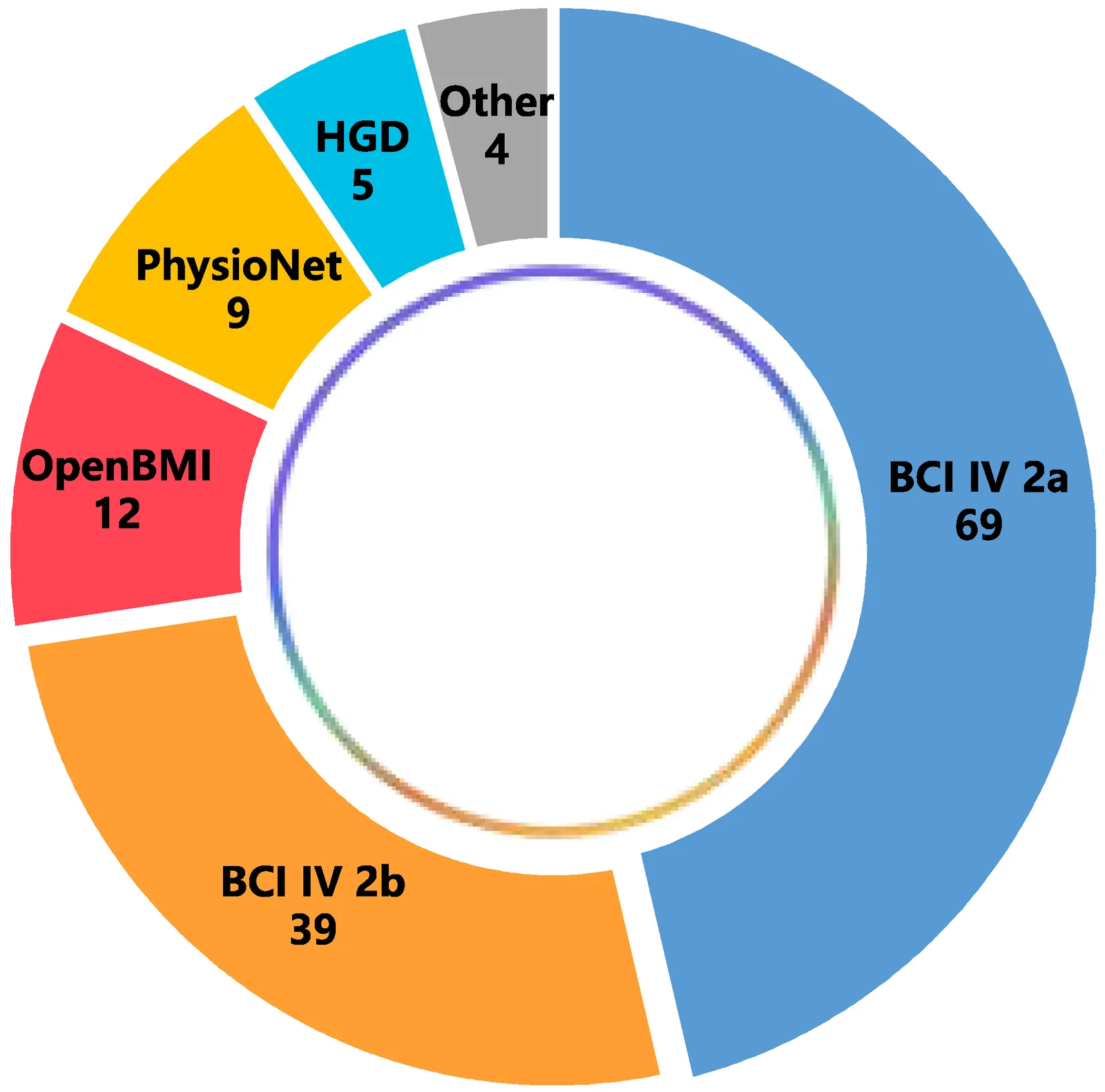
Frequency of each dataset in the selected studies.

In this study, we review studies that utilize several public MI datasets, including datasets from BCI competitions and specified institutions. The datasets are obtained from either the BCI-specific websites or specific search engines for datasets. Table 1 summarizes the key details of the datasets used in the studies in this paper. Moreover, Figure 5 indicates the number of times a dataset is used in the selected studies.

Some of the common datasets in our selected studies are the following:

- BCI Competition IV Dataset 2a: This dataset was recorded at a sampling frequency of 250 Hz from nine healthy participants. Four MI tasks, i.e., movement of left hand, right hand, both feet, and tongue, were performed by all the subjects and the corresponding data was recorded using 22 electrodes placed on the subject’s scalp. This dataset is the most widely used across our reviewed literature, not only because it was recorded with utmost precision, but also due to its four discriminant tasks that allow a more rigorous comparison of architectures compared to the binary datasets. Moreover, this dataset has been available since 2008, which means new models can be benchmarked directly against a large number of prior reported architectures using this dataset.
- BCI Competition IV dataset 2b: Unlike dataset 2a, dataset 2b of the BCI Competition IV was recorded using only three electrodes (Cz, C3, C4) and the MI tasks were restricted to the movement of the left and right hands only. The number of subjects and sampling frequency remains the same as dataset 2a. It is the second most widely used dataset in our reviewed studies due to the overall high precision of the acquired signals in BCI Competition IV. The dataset was recorded in a highly controlled environment and the participants were given thorough guidelines before acquiring the signal, making the signals highly relevant to the MI tasks performed.
- OpenBMI dataset: This dataset was recorded from 54 healthy subjects performing left/right-hand MI tasks and the signals were acquired using 62 electrodes. Despite having a large number-of-subjects count that might be favorable for subject-independent or cross-subject evaluation, this dataset is less frequently used in our reviewed studies; this might be due to its relatively recent release or its high channel count increasing the computational overhead and preprocessing costs.
- PhysioNet: The PhysioNet dataset was recorded from 109 healthy subjects performing four MI tasks and the dataset was recorded using 64 EEG channels. Despite being one of the largest public MI datasets, its use in BCI studies is less frequent compared to the BCI IV 2a and 2b datasets, which may be credited to its single-session protocol and limited per-subject trial count compared to two sessions (on different days) with 288 trials each in the BCI IV 2a dataset. This may cause overfitting and deep architectures might struggle to achieve reasonable accuracy; hence, studies validating architectures on this dataset remain limited.
- High Gamma Dataset (HGD): The HGD dataset was recorded from 14 healthy subjects performing four MI tasks using 128 electrodes. This dataset is used less often in validating MI-BCI frameworks as it has higher computational complexity due to the large number of EEG channels, long recordings, and large data volume. Moreover, due to the dominance of the BCI Competition IV datasets in validating MI classification architectures, HGD naturally is a less frequent choice for benchmarking MI-BCIs.
- Others/private: Other than these publicly available datasets, some studies validate their frameworks on privately collected data. While this allows researchers to tailor acquisition protocols to specific hypotheses, results reported on privately collected datasets cannot be compared against public benchmarks directly. In addition, acquiring such datasets requires a lot of expertise; therefore, more than 90% of the studies in the literature use publicly available MI-BCI datasets.

## 4. MI-BCI Classification Overview

**Figure 6:**
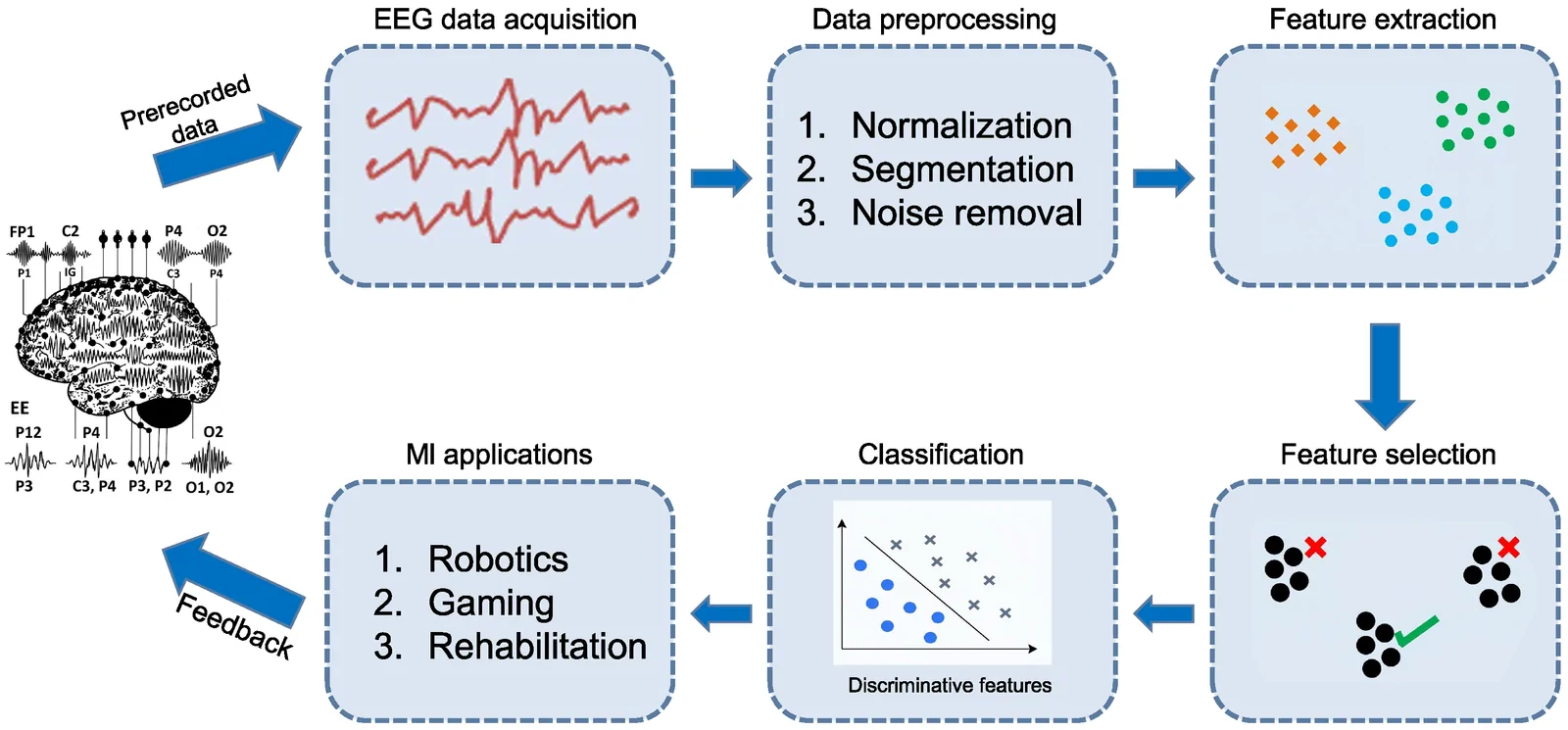
Schematic overview of the MI-BCI classification framework classification framework overview.

Figure 6 represents a brief overview of a standard MI-BCI classification framework. A typical MI-BCI classification framework consists of an EEG data acquisition stage, a data preprocessing stage, a feature extraction stage, a classification stage, and an application stage. These stages can be explained in the following steps.

1. The first step is data acquisition. Most studies in the literature use publicly available datasets, such as datasets from BCI Competitions.
2. In the next step, the data is usually preprocessed to remove artifacts and obtain the desired frequency range. Band-pass filtering is commonly employed to extract signals of the desired frequency. In general, a frequency range between 4 and 30 Hz is considered relevant for MI tasks. Moreover, certain studies use noise removal techniques, such as MSPCA or ICA, or manually remove noise using visualization techniques.
3. Next, a feature extraction technique is designed to extract the local and global patterns of the EEG data. The extracted features contain inherent patterns of the EEG data as well as time-dependent features corresponding to the MI tasks.
4. In the final step, a classifier is used to classify the extracted features into their respective MI classes. Common classifiers include FFNN, SVM, ELM, and LDA.
5. The designed frameworks are evaluated on various publicly available MI datasets using metrics such as accuracy, kappa value, and F1 score.
6. Moreover, to verify the effectiveness of the proposed model, the performance is compared with state-of-the-art models, such as EEGNet, DeepConvNet, or ShallowNet.
7. Statistical tests such as the t-test, z-test, ANOVA, and Wilcoxon test are used to determine whether the observed superiority of the proposed technique compared to the established benchmarks and existing studies is statistically significant or occurring by chance.
8. Once a framework has passed all the evaluation criteria, it is potentially ready to be used in BCI applications.

## 5. Sequence and Generative Models

In this section, different sequence-oriented, attention-based, hybrid, and generative models used for MI classification are discussed. Sequence-oriented models include RNNs, LSTMs, GRUs, temporal CNN-RNN hybrids, and transformers. Attention-based models include transformer encoders and CNN-attention architectures, whereas generative models include GANs, VAEs, and autoencoders used for reconstruction, representation learning, or data augmentation.

### 5.1. Recurrent Neural Networks (RNNs)

RNNs are one of the most renowned types of neural network; they are specifically designed to handle sequential data, such as time series or NLP. In the context of MI classification, RNNs can play a crucial role as EEG data is inherently time-dependent and can take advantage of sequential processing of RNNs to extract time-dependent features. Unlike traditional CNNs, which apply a single fixed-kernel convolution uniformly across the entire EEG sequence, RNNs maintain a shared hidden state, updated at each time step, allowing information from previous time steps to be retained, which can be very favorable in MI-BCI applications.

The architecture of RNNs can be defined by the following equations:

(1) $$y_{t}=g\left(W_{y}h_{t}+b_{y}\right)$$

where $y_{t}$ is the output of RNN at time *t*, $W_{y}$ is the output weights and $b_{y}$ is the added bias. $h_{t}$ is the hidden state at time *t*, which is given by

(2) $$h_{t}=f\left(W_{h}h_{t-1}+W_{x}x_{t}+b_{h}\right)$$

where *f* represents the activation function applied, which is usually ReLU or tanh.

The RNN’s learning process is based on the loss function, which computes the difference between the predicted and actual output. Based on the loss value, the weights are updated using backpropagation through time (BPTT) to minimize the loss function. However, if the EEG sequence is very long, backpropagation requires gradient calculations in several time steps, which can potentially lead to vanishing or exploding gradients. This led to the development of more advanced models such as LSTMs, GRUs, and bidirectional RNNs.

#### 5.1.1. Long Short-Term Memory (LSTM)

LSTMs are a class of neural networks designed to solve or avoid the vanishing or exploding gradient problems in conventional RNNs by proposing a gating mechanism and a memory unit. This memory unit holds the information in long-sequence data without information change. Moreover, LSTMs use three types of gates: the input gate, output gate, and forget gate. These gates together manage the flow of information into and out of each cell in the sequence.

The gating mechanism in LSTMs is particularly useful in MI-BCIs as the event-related desynchronization/synchronization (ERD/ERS) patterns in MI data unfold gradually, so the gating mechanism allows the network to retain the task-relevant temporal features while suppressing artifacts and noise. However, LSTMs can be helpful mainly in retaining temporal features, and spatial representations need to be captured separately, which is why a convolutional module is usually coupled with LSTM-based architectures to exploit maximum information from the MI-EEG signals.

The functioning of LSTMs can be explained in the following steps.

At the beginning, the LSTM cell is initialized with $C_{0}$ as the initial state and $h_{0}$ as the hidden state.

Each feature vector $x_{t}$ of EEG data at time *t* is fed to the LSTM. The forget gate at this point determines which information is to be retained from the previous time step. This helps in retaining the significant information and getting rid of any noise or irrelevant signals. The forget gate $F_{t}$ can be represented as

(3) $$F_{t}=f\left(W_{f}\cdot \left[h_{t-1},x_{t}\right]+b_{f}\right)$$

Next, the input gate manages to add new information, and this new information, along with the information from the previous state, is managed by the cell state, given as

(4) $$C_{t}=f_{t}⊙C_{t-1}+i_{t}⊙{\tilde{C}}_{t}$$

where

(5) $$i_{t}=f\left(W_{i}\cdot \left[h_{t-1},x_{t}\right]+b_{i}\right)$$

(6) $${\tilde{C}}_{t}=\tanh\left(W_{c}\cdot \left[h_{t-1},x_{t}\right]+b_{c}\right)$$

Here, $i_{t}$, *f*, and ${\tilde{C}}_{t}$ represent the input gate, the activation function, and the candidate cell state, respectively.

The output gate decides which part of the input should be retained by storing it in the hidden state at this time step. This ensures that the key information of the sequence is retained. The output gate $O_{t}$ can be defined as

(7) $$O_{t}=f\left(W_{o}\cdot \left[h_{t-1},x_{t}\right]+b_{o}\right)$$

Finally, the hidden state is determined using the following equation:

(8) $$h_{t}=O_{t}⊙\tanh\left(C_{t}\right)$$

This hidden state contains the key features extracted from the EEG data at this time step. During the training of the model, the hidden state is updated using backpropagation based on the loss between the actual and predicted values.

#### 5.1.2. Gated Recurrent Unit (GRU)

GRUs work on the same concept as LSTMs; however, they offer a simplified architecture by proposing an update gate, which is a combination of the forget and input gates. This allows GRUs to retain information in long-sequence data while being more computationally efficient compared to LSTMs. GRUs have two primary gates: the reset gate and the update gate. These gates together decide the amount of new information to be stored and the information to be retained from the past.

The reset gate $r_{t}$ is responsible for forgetting past information, thus allowing the network to reset the memory for the new input in the sequence. The update gate $z_{t}$ then controls the new information and a portion of past information, creating a balance between learning new information and memorizing past information.

GRUs offer the same benefit as LSTMs for MI-EEG, with their gating mechanism able to retain temporal dependencies in EEG data; however, their reduced parameter count compared to LSTMs makes them an even better choice for MI-BCIs, as MI-BCIs are required to be computationally efficient for real-time applications.

The reset gate and update gates are mathematically defined as

(9) $$r_{t}=f\left(W_{r}\cdot \left[h_{t-1},x_{t}\right]+b_{r}\right)$$

(10) $$z_{t}=f\left(W_{z}\cdot \left[h_{t-1},x_{t}\right]+b_{z}\right)$$

The reset gate modulates the candidate hidden state using the previous hidden state and the new input, given by

(11) $${\tilde{h}}_{t}=\tanh\left(W_{h}\cdot \left[r_{t}⊙h_{t-1},x_{t}\right]+b_{h}\right)$$

Finally, the hidden state is modulated by the update gate using the previous hidden state and the candidate hidden state as follows:

(12) $$h_{t}=\left(1-z_{t}\right)⊙h_{t-1}+z_{t}⊙{\tilde{h}}_{t}$$

#### 5.1.3. Bidirectional LSTMs

Bidirectional LSTMs offer context from both the past and the future by processing sequential data in a bidirectional manner. Bidirectional RNNs enhance traditional RNNs by making connections in both the forward and backward directions, thus allowing a more thorough understanding of the input sequence. Standard bidirectional RNNs consist of two distinct RNN layers to process information in the forward and backward directions.

The forward RNN $h_{t}^{\left(f\right)}$ processes the input from the beginning toward the end, given by

(13) $$h_{t}^{\left(f\right)}=\mathrm{RNN}\left(x_{t},h_{t-1}^{\left(f\right)}\right)$$

Conversely, the backward RNN $h_{t}^{\left(b\right)}$ processes the sequence in the reverse direction, from the end to the beginning, as follows:

(14) $$h_{t}^{\left(b\right)}=\mathrm{RNN}\left(x_{t},h_{t+1}^{\left(b\right)}\right)$$

The final hidden state is obtained by combining the forward and backward states as follows:

(15) $$h_{t}=\left[h_{t}^{\left(f\right)},h_{t}^{\left(b\right)}\right]$$

Bidirectionality can be helpful in offline MI-BCI evaluations, where the network has both past and future context available to localize ERD/ERS onset. However, this reliance on future time steps can be a limitation in online BCIs, making them less adopted architectures for real-time BCIs.

### 5.2. Variational Autoencoders (VAEs)

A VAE is a type of generative model that is based on the encoder–decoder architecture. The encoder learns hierarchical and time-dependent features from the data and converts them into a compressed latent representation. The decoder then uses that latent representation to reconstruct the original input data from the compressed representation. However, instead of generating a single output, the encoder produces a probability distribution of a parameter over the latent variables, given by

(16) $$q\left(z|x\right)=N(z;\mu \left(x\right),\sigma^{2}\left(x\right))$$

where *x* and *z* represent the input signal and latent variable, respectively, and N indicates that the distribution of data is Gaussian.

The decoder is designed to approximate the original input by sampling from the latent distribution provided by the encoder. The decoder is defined as

(17) $$p\left(x|z\right)=N\left(x;\hat{x},\sigma^{2}\right)$$

VAEs are trained to learn the data patterns by minimizing the loss function, which is a combination of reconstruction loss from the decoder and Kullback–Leibler (KL) divergence. The reconstruction loss is a measure of how well the decoder has reconstructed the original input and is given by

(18) $$\mathrm{Reconstruction}\;\mathrm{Loss}=-E_{q(z|x)}\left[\log p\left(x|z\right)\right]$$

where $E_{q}$ indicates the expected value with respect to the distribution, and the negative sign indicates that the goal of training is to minimize this value.

Moreover, the KL divergence helps in learning meaningful features in the latent representation by regularizing it to follow a standard normal distribution, as given by

(19) $$\mathrm{KL}\;\mathrm{Divergence}=D_{\mathrm{KL}}\left(q\left(z|x\right)\|\;p\left(z\right)\right)$$

where $D_{\mathrm{KL}}$ indicates the KL divergence from q(z∣x) to p(z).

The probabilistic latent space in VAEs is well suited for MI-BCIs as it offers a structured way to model the inter-subject variations in EEG data rather than treating them as noise, which is why they are used for both subject-invariant representation learning and data augmentation to solve the data scarcity challenge in EEG data.

### 5.3. Generative Adversarial Networks (GANs)

GANs are a category of generative models that employ adversarial training to generate new, realistic data samples from a predetermined training set. GANs consist of two neural networks, the generator and the discriminator, which are trained competitively. The generator aims to generate synthetic data samples from random noise, whereas the discriminator aims to differentiate between real data (from the training set) and the synthetic data generated by the generator.

As most public EEG datasets have a limited dataset size, GANs can be promising for generating synthetic data closely mimicking the real EEG data. Moreover, the adversarial training in GANs does not need an explicit likelihood model of the EEG signal, making them well suited for synthetic EEG data generation without assuming a particular parametric noise model.

The training process for GANs can be expressed by the following loss functions:

(20) $${\mathrm{Loss}}_{D}=-E_{x∼p_{\mathrm{data}}}\left[\log D\left(x\right)\right]-E_{z∼p_{z}}\left[\log\left(1-D\left(G\left(z\right)\right)\right)\right]$$

(21) $${\mathrm{Loss}}_{G}=-E_{z∼p_{z}}\left[\log D\left(G\left(z\right)\right)\right]$$

In this context, ${Loss}_{G}$ and ${Loss}_{D}$ represent the generator and discriminator loss, respectively. D(x) represents the discriminator’s probability that *x* is a real data sample, and G(z) represents the synthetic data generated by the generator using noise *z* sampled from a random distribution $p_{z}$.

The discriminator is trained to minimize the loss function described in Equation (20), i.e., to maximize its ability to correctly discriminate real EEG samples from synthetic ones, whereas the generator is trained to generate synthetic samples as realistic as possible (i.e., to maximize the discriminator loss). However, in this classical minimax formulation, the early training steps might produce very weak gradients for the generator, as the discriminator can easily identify fake samples. Therefore, most GAN-based studies instead train the generator to minimize the loss function in Equation (21) as a separate, non-saturating loss, leading to stronger gradients even in the early stages of training. Training proceeds with alternating updates from the generator (minimizing ${\mathrm{Loss}}_{G}$) and discriminator (minimizing ${\mathrm{Loss}}_{D}$) until the discriminator is no longer able to correctly distinguish the generator’s generated samples from the real EEG samples.

A well-known failure mechanism in GANs is mode collapse, where the generator, instead of capturing the complete variability of the real data, learns to produce patterns identical to only a subset of the data. This can be detrimental in MI classification, since a collapsed GAN might create data augmenting only a narrow set of EEG patterns, without reflecting the natural inter-subject and inter-trial variations of the EEG recordings, potentially leading to exaggerated downstream classification performance without actual improvements in terms of generalization capability. Mode collapse can be assessed via quantitative methods, such as the Frechet inception distance (FID) or the maximum mean discrepancy (MMD) between real and synthetic distributions, or qualitative methods, such as visual inspection of intra-class variance comparison or monitoring training logs to see if the discriminator loss approached zero in the early stages of training, which can be a classic indicator of mode collapse in generators.

### 5.4. Transformers

**Figure 7:**
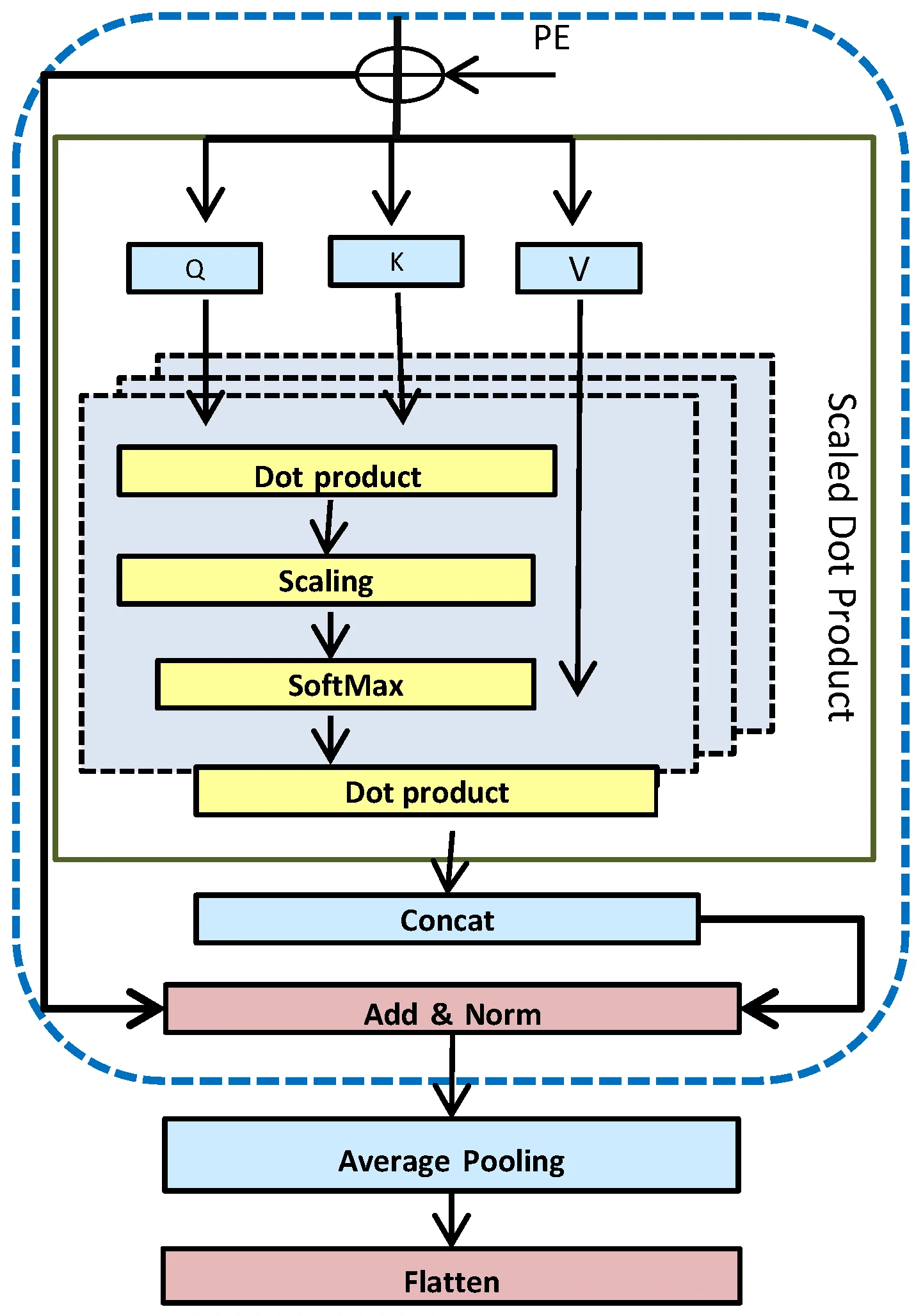
Transformer architecture with self-attention at its core.

Transformers are by far the most innovative class of sequence models and have significantly changed the DL paradigm and addressed the limitations of traditional sequential models like RNNs and LSTMs. The success of transformers lies in the novel architecture, having a self-attention mechanism at its core, as shown in Figure 7, which assesses the relationships between each token in the input sequence to every other token using queries (Q), keys (K), and values (V) as its three main vectors.

This can be well suited to MI-EEG data as it can directly model the dependencies between distant channels or time points in an EEG sequence, compared to RNNs that can only model dependencies between recent time points in a sequence. However, self-attention alone lacks local inductive bias, which may limit the full exploitation of the MI-EEG data, which is why most top-performing transformer-based architectures in the literature couple them with convolutional or temporal–convolutional modules to extract maximum feature representation from the EEG data.

For a given EEG input, the Q, K, and V vectors are computed using the following equations:

(22) $$XW^{Q}=Q,\;XW^{K}=K,\;XW^{V}=V$$

In this case, the input matrix *X* contains embeddings for every token, whereas the learning weight matrices $W^{Q}$, $W^{K}$, and $W^{V}$ translate the input into query, key, and value spaces, respectively. During training, the self-attention mechanism learns to score each token based on how much attention it should pay to other tokens in the input sequence. In order to ensure consistent gradients during training, the attention score is calculated by taking the dot product of the Q and K vectors and normalizing it by the square root of the K vector’s dimension, $d_{k}$. This produces an attention score matrix that is then normalized using the softmax function and finally multiplied by the V vector to attain an attention value.

(23) $$\mathrm{Attention}\left(Q,K,V\right)=\mathrm{softmax}\left(\frac{QK^{T}}{\sqrt{d_{k}}}\right)V$$

The softmax function ensures that the attention weights for each token add up to 1, thus creating a valid probability distribution. In order to attend to different parts of the input sequence from many perspectives simultaneously, multi-head attention is employed, which computes the attention score concurrently across multiple heads. The final attention score is computed by concatenating the output from multiple heads, as given by

(24) $$\mathrm{MultiHead}\left(Q,K,V\right)=\mathrm{Concat}\left({\mathrm{head}}_{1},{\mathrm{head}}_{2},\ldots ,{\mathrm{head}}_{h}\right)W^{O}$$

where the computation of each attention head is

(25) $${\mathrm{head}}_{i}=\mathrm{Attention}\left(QW_{i}^{Q},KW_{i}^{K},VW_{i}^{V}\right)$$

Several self-attention layers combined constitute the encoder of the transformers. Each layer executes self-attention independently to identify various patterns of the EEG data. The original transformer architecture also includes a decoder, which generates the output by using the encoded input data from the encoder and the sequence of previously generated outputs it produced. The decoder is trained auto-regressively, i.e., it can only attend to the past tokens it generated, and the cross-attention mechanism allows it to attend to specific parts in the input relevant to generating the new output token. However, since MI decoding is a sequence-to-label classification task instead of a sequence-to-sequence generation task, most MI classification studies use only the encoder component of the transformer architecture. A classification layer is used after the self-attention-based encoder to classify the features into the MI tasks.

Since transformers are not inherently aware of the sequence of tokens, positional encoding is employed to incorporate the positioning information of the input sequence. These encodings are typically generated using the sine and cosine functions as follows:

(26) $$\begin{array}{cc} {\mathrm{PE}}_{\left(pos,2i\right)} & =\sin\left(\frac{pos}{10000^{2i/d_{model}}}\right)\\ {\mathrm{PE}}_{\left(pos,2i+1\right)} & =\cos\left(\frac{pos}{10000^{2i/d_{model}}}\right) \end{array}$$

where pos indicates the position of the token, *i* represents the dimension of encoding, and *d* is the total dimensionality.

A practical limitation in the traditional transformer architecture is the quadratic time and memory complexity of the standard self-attention mechanism, as shown in Equation (23), because every time point attends to every other time point. In EEG, a single MI trial has thousands of raw samples. When channels and time points are flattened into a token sequence, the computational cost scales quadratically, which motivates the use of efficient transformer variants. They are widely used in the DL literature and have begun to appear in EEG decoding as well. Swin Transformer [58], for instance, uses windowed attention, which restricts attention to local windows that are periodically shifted across layers, reducing complexity to scaling roughly linearly with the sequence length. Other efficient attention families include linear attention approximations (e.g., Performer [59], Linformer [60]), which approximate the softmax attention kernel to achieve linear complexity.

## 6. Discussion

In this section, we discuss the selected studies and, based on their data, we find answers to the research questions raised in this study. Moreover, this section highlights some key challenges in MI decoding and potential solutions for achieving high performance. All abbreviations used are defined in the Abbreviations section at the end of the article.

### 6.1. Analysis of the Compared Studies

For a comparative analysis, we selected 78 unique studies employing sequence, attention-based, hybrid, and generative models for MI classification, and the comparative analysis is presented in Table 2, Table 3 and Table 4. Because individual studies differ in MI tasks, preprocessing strategies, validation design, dataset choice, and various other hyperparameters, their reported accuracies cannot be compared directly and replicating individual studies under matched hyperparameters is also beyond the scope of this study. Therefore, our analysis treats the statistics reported from the studies as an unweighted descriptive synthesis rather than a formal meta-analysis. This distinction is fairly important because each evaluation protocol has a different difficulty level, and therefore a high accuracy without considering the evaluation protocol cannot directly be interpreted as a better architecture. It could also reflect an easier experimental setup, better preprocessing, data augmentation, a favorable validation split, or target–subject calibration. Therefore, direct comparisons in this review are only made for studies using the same dataset and evaluation protocol to ensure that a thorough architectural comparison is possible.

**Figure 8:**
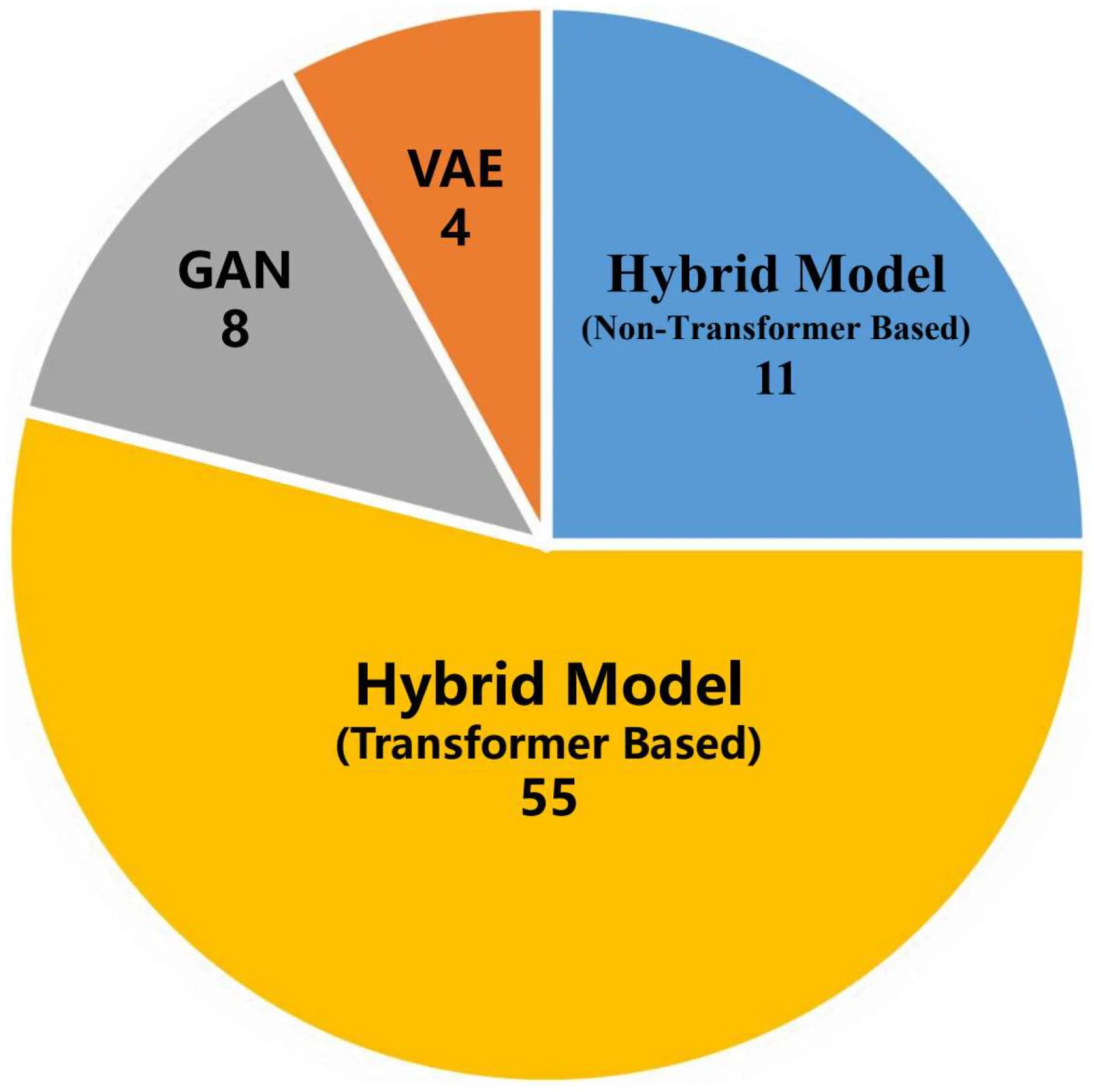
Frequency of different sequence, attention-based, hybrid, and generative models utilized across selected studies.

As shown in Figure 8, the model categories are not mutually exclusive. A transformer- or RNN-based architecture is typically layered on top or in parallel with a convolutional module for better feature extraction, and a GAN or VAE architecture is usually coupled with a convolutional classifier for high classification accuracy. Most of the reviewed studies use hybrid models that are a combination of different sequence, generative, or convolutional networks. Moreover, the literature is heavily concentrated on the BCI IV 2a dataset, with 69 out of 78 studies using this dataset to benchmark the results, as shown in Figure 5; whereas the OpenBMI dataset, which could be a much better option, especially for validating subject-independent architectures, is only used in 12 studies. This strong imbalance may be a cause of the high accuracy reported on BCI IV 2a, which might be due to extensive tuning to this particular dataset rather than being due to better EEG generalization, which could limit how far the analysis drawn from these studies can be extended beyond this particular dataset.

Based on an analysis of the reviewed studies, the following questions are addressed: (1) Which models produced the best MI classification results? (2) To what degree have sequence, attention-based, and generative models progressed over time in achieving subject-independent classification? (3) To what extent and in which capacity have generative models contributed to MI classification? (4) Is there a definitive correlation between a model’s performance and its complexity?

#### 6.1.1. Which Architectures Produced the Best MI Classification Results?

Table 2 lists the 11 non-transformer CNN-RNN architectures reviewed in this study, whereas Table 3 lists the 55 transformer- and attention-based architectures. The first insight drawn from these tables is that almost all high-performing architectures use a combination of multiple networks. Most sequence model-based designs are paired with a convolutional or temporal convolutional module to provide the local inductive bias that sequential models lack. Secondly, the non-transformer-based models (RNNs, LSTMs, GRU families) are concentrated almost entirely in the earlier studies between 2018 and 2022, whereas transformer and attention-based architectures dominate the literature from 2021 onward. This research shift is not only dominant in the EEG signal classification domain, but also in the NLP and vision domains.

This architectural shift, however, does not necessarily reflect a proportional gain in accuracy. For instance, looking into the BCI IV 2a accuracies under the SD protocol, it can be noticed that the late CNN-LSTM-based architectures already reported accuracies in the range of 80–88%, for example, [43,63] achieve an accuracy of 87.68 and 87.14%, respectively, whereas the recent transformer-based frameworks span a wide range, between 75 and 90%, with most architectures clustering in the 82–85% range [47,70,73,76,79]. Therefore, it can be observed that the reported ceiling has not moved substantially with the shift to transformer-based designs.

However, what has evidently changed is the parameter efficiency at which those accuracies are achieved. Some transformer-based architectures have reached accuracies in the 85–88% range with an order of magnitude fewer parameters compared to the earlier deep CNN-LSTM-based frameworks. For example, TriNet [105] achieved 87.30% accuracy with only 18K parameters, compared to MBHNN’s [65] 86.15% accuracy with 521 K parameters. Moreover, transformer-based approaches have also shown a consistent edge over the RNN/LSTM baselines in multi-class, cross-subject classification. While most of the RNN/LSTM-based designs focused on achieving a high within-subject classification performance, transformers, with their global attention mechanism, focused on extracting long-range dependencies and global patterns, enabling a more generalized feature representation that is less sensitive to inter-subject variations (discussed in more detail in Section 6.1.2). Therefore, the advantage of the transformer-based architectures in this review is better characterized as a gain in accuracy-per-parameter and in modeling long-range dependencies rather than an apparent peak in accuracy.

#### 6.1.2. To What Degree Have Sequence and Generative Models Progressed over Time in Achieving Subject-Independent Classification?

Subject-independent classification has always been a bottleneck in MI-EEG decoding due to inter-subject variations arising from physiological (skull thickness, cortical folding, etc.), cognitive (BCI literacy), or session/hardware (cap placement, impedance drift, etc.) differences. In order to analyze the progress of sequence and generative models over the years in subject-independent classification, we compare some of the studies published over the years on BCI Competition IV dataset 2a under the SI evaluation protocol.

**Figure 9:**
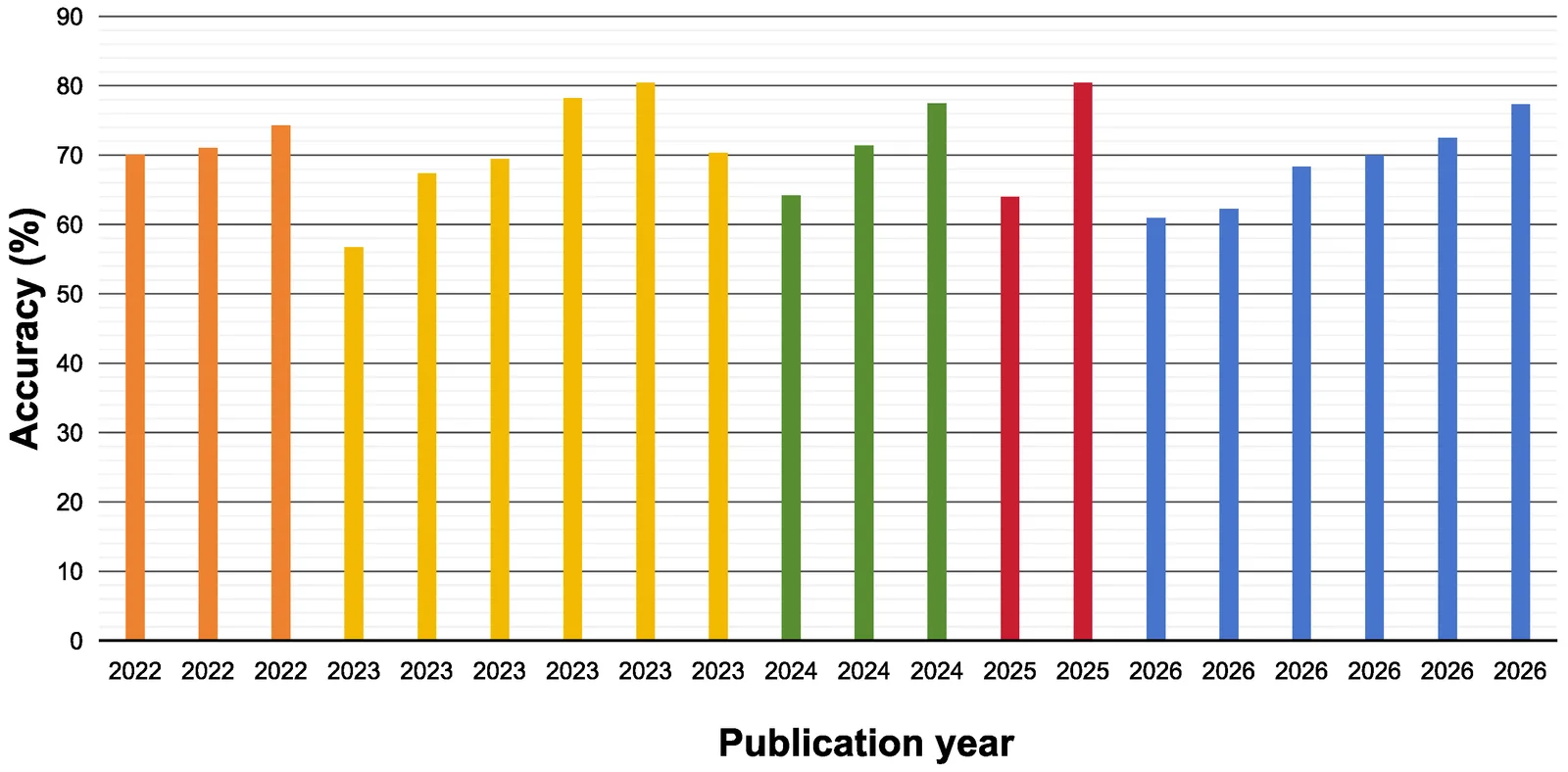
Analysis of subject-independent performance over the years on BCI IV-2a dataset. The studies represented in the figure are cited as [46,58,67,75,76,79,81,83,84,86,89,97,98,103,105,111,113,114,119,120].

As shown in Figure 9, subject-independent classification has only been given attention in recent years, between 2022 and 2026, and the accuracy is less than 80% in most of the studies, despite the high amount of research work published, as demonstrated in Figure 9. Most of the studies have accuracies hovering around the 75–77% range, which shows that subject-independent classification is still a significant challenge. However, it can be observed that architectures such as st-CViT [84] and CNNViT-MILF-a [103], that are explicitly designed for subject-independent classification, report more consistent accuracy gains compared to others, suggesting that explicit alignment strategies can be more effective for generalization to unseen data.

It is worth mentioning that the reported accuracies in the literature are also obtained under highly controlled and ideal experimental conditions and do not necessarily imply robust transferability to real-world applications, where the acquired signals are affected by variations in the attention spans of subjects, individuals’ fatigue levels, contact with electrical equipment, and other environmental factors that are not tightly controlled. Therefore, a further degradation in performance can be expected when these architectures are deployed in real-world settings. For BCIs to be scalable and generalized, subject-independent classification performance needs further improvement.

#### 6.1.3. To What Extent and in Which Capacity Have Generative Models Contributed in MI Classification?

VAEs and GANs are generative models that have mainly contributed to MI classification over the years in data generation and reconstruction, as shown in Table 4. A noticeable gain of about 7% is observed in [121] when 25% of the training data was generated using a VAE. However, it is observed that the performance deteriorated when 100% of the data were generated using VAEs, demonstrating that while VAEs can be effective for data augmentation, they should be used in moderation, to supplement the original data but not to replace it completely. A similar increase in accuracy, ranging from 5% to 18%, is observed in [122,123,124,125,126,127,128], where GANs and VAEs are used for data augmentation and a substantial gain in performance is observed. The gain is more pronounced in the studies where the baseline accuracy was low and generative models had a huge margin to improve, such as in CS-GAN, where the accuracy increased from 52.12% to 67.97%. A downstream classifier is usually used along with these generative models to classify the user’s intent into the MI tasks, and the overall performance depends heavily on the classifier performance along with the quality of the augmented data.

Moreover, HiFi-GAN [129] demonstrates another promising application of GANs in MI classification, where it is used to accurately synthesize missing or corrupted channel data. In [130], a VAE is used to disentangle task irrelevant feature to enhance feature separation and improve MI decoding performance. Overall, in most of the reviewed studies, VAEs and GANs are mostly used to generate synthetic data that closely mimics the original EEG data.

It is worth noting that the performance of these data augmentation networks cannot be assessed directly via the downstream classification accuracy without considering the neurophysiological validity of the augmented data. This is an important distinction, since a synthetically generated signal might improve the classification accuracy of an MI decoding task without faithfully reproducing the neurophysiological characteristics of real EEG data. A neurophysiologically valid EEG signal should demonstrate accurate ERD/ERS in the mu and beta bands and the power spectral density distribution should reproduce the original EEG data distribution. Moreover, the augmented signals should exhibit spatial patterns and channel correlations consistent with the actual brain topography and should demonstrate a closely matching distribution to the true EEG data in both the feature and latent spaces.

In [124], spectrograms of real and synthetic data are compared to analyze the neurophysiological validity of the augmented data. In [126], real and fake data samples are compared in the time domain, and in [128] they are compared in the time–frequency domain. Moreover, in [51] the spatial distribution of real and synthetic data is compared for neurophysiological analysis. These studies demonstrate that generative models have been effective in generating synthetic data closely resembling true EEG data and have shown promising results in enhancing MI decoding performance.

**Table 4:** Within-family comparison of GAN- and VAE-based studies by generative role. Reported values are complete-pipeline outcomes (generative component + downstream classifier) and are compared only within the same study or the same generative role, not pooled across roles.

Reference	Year	Model Architecture	Protocol	Methodology
[126]	2020	GAN	Role: Sample augmentation; results: baseline: 73.04%, after augmentation: 80%.	Data augmentation is achieved using a classical GAN network and a standard CNN is used for classification.
[125]	2021	CycleGAN	Role: Data augmentation; results: baseline: 60% (raw), after augmentation: 78.3%.	Cycle-GAN is used to mimic the EEG spectral topographies of the stroke patients, hence expanding the number of training samples.
[127]	2021	LGAN	Role: Data augmentation; results: baseline: 74.65% (raw), after augmentation: 83.996%.	Combines LSTM and GAN networks to create synthetic EEG samples for enhanced generalization.
[124]	2021	CS-GAN	Role: CS sample augmentation; results: baseline: 52.12%, after augmentation: 67.97%.	Dataset size is increased using a CSP-based GAN framework, thus targeting the CS data scarcity problem.
[51]	2022	Channel-wise VAE	Role: Reconstruction/inter-task transfer; results: 0.83 (private dataset); 0.69 (BCI IV 2a).	A channel-wise VAE is used for signal reconstruction and inter-task transfer learning, and the results are evaluated on an external dataset.
[128]	2024	WGAN-GP	Role: Time–frequency sample augmentation; results: baseline: 80.2% (raw), after augmentation: 83.3%.	A framework consisting of Wasserstein GAN with gradient penalty is designed to generate time–frequency representations of EEG for augmentation-based classification.
[131]	2024	GITGAN	Role: CS domain adaptation; results: 84.0% (OpenBMI); 82.9% (binary 2a); 69.2% (four-class 2a).	A generative inter-subject transfer framework is developed using adversarial adaptation and an effective source-data selection method.
[121]	2025	EEG-VAE	Role: Data augmentation; results: baseline: 0.76; augmented data: 0.83 at 25%, 0.80 at 100%.	VAE is used to generate synthetic data closely resembling original data to improve the feature-learning ability of the downstream classifier.
[122]	2025	REVG (VAE+WGAN)	Role: Reconstruction and data augmentation; results: raw 75.40%; baseline: 75.40%, VAE + WGAN: 76.83%.	Combines VAE-based reconstruction with WGAN-based augmentation to increase the generalization capability of the model.
[123]	2025	MINDGAN	Role: Data augmentation; results: baseline: 73.26, with augmentation: 85.38%.	Deep convolutional GAN conditioned on class labels generates high-quality EEG data to solve the problem of data scarcity paired with a specialized hybrid classifier.
[129]	2026	HiFi-GAN	Role: Channel synthesis; results: baseline mean: 58.91%; mean after channel synthesis: 60.72%; gains up to 7%.	Synthesizes additional EEG channel to enhance data size and improve decoding performance.
[130]	2026	Dual-guided iVAE	Role: Feature separation; results: cross-session: 79.02%; CS: 66.06%.	VAE is used to learn semantically distinguished feature representations of the EEG data to reduce the inter-subject variation challenge.

#### 6.1.4. Is There a Definitive Correlation Between a Model’s Performance and Its Complexity?

From a theoretical perspective, it is evident that a deeper architecture can enhance a model’s ability to extract intricate patterns of EEG data; however, a very deep architecture with several learnable parameters might also lead to overfitting, especially in small datasets such as BCI IV 2a. This necessitates the need to analyze whether there is a linear correlation between the model’s computational cost and performance, or whether shallow architectures can also perform well given the right structure.

**Figure 10:**
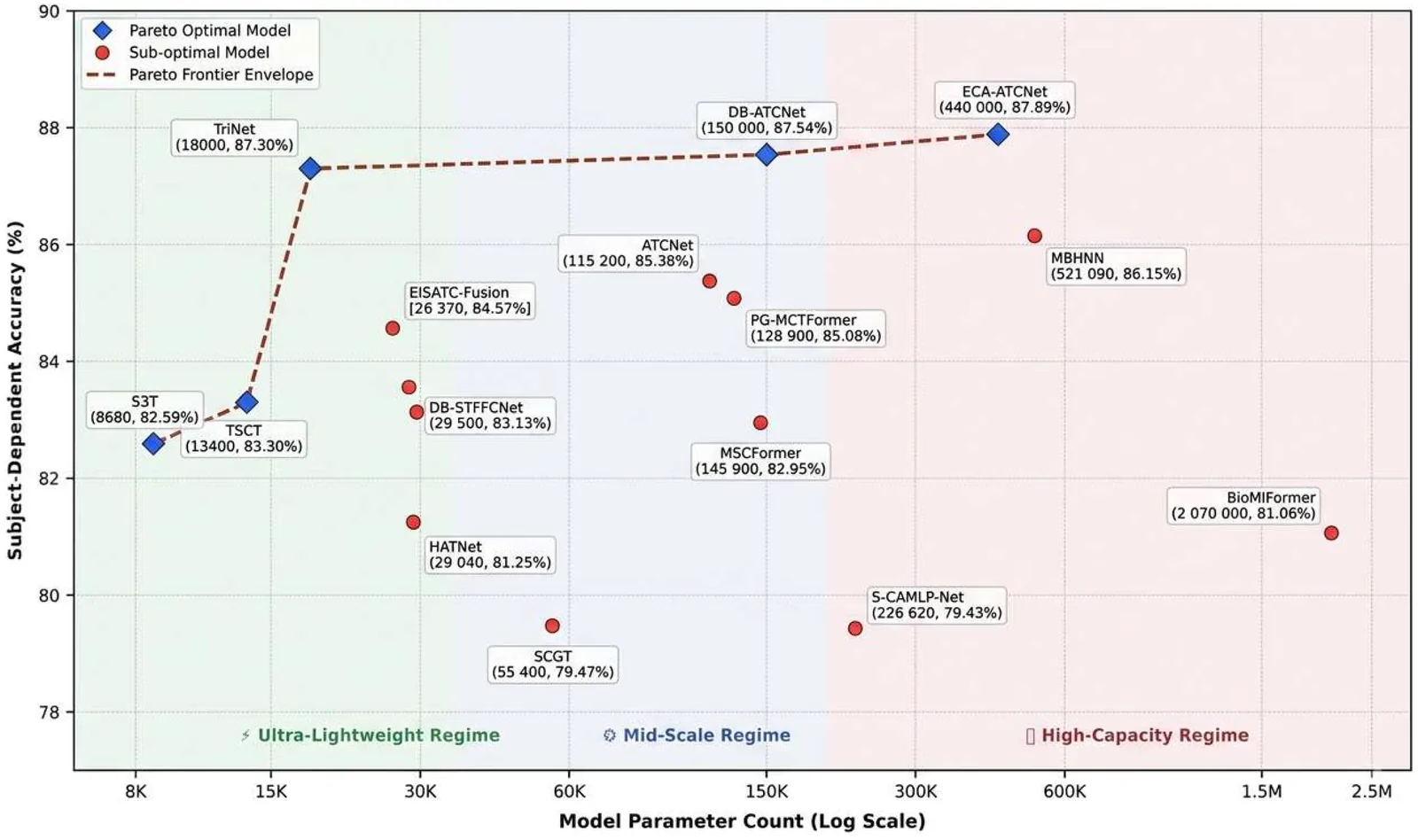
Parameters vs. accuracy plot on BCI IV 2a under the SD protocol.

It should be noted that most of the studies in this review do not explicitly report their parameter count or computational cost; therefore, our analysis is drawn from only a subset of the 78 reviewed studies. Table 5 lists the number of trainable parameters, FLOPs, inference time, and training time along with accuracy on the BCI IV 2a dataset, and Figure 10 plots a direct comparison of the accuracy (BCI IV 2a in the SD evaluation protocol) with the number of parameters.

It can be observed that there is no apparent correlation between accuracy and the number of parameters. Several architectures, such as S3T [47], TriNet [105], and TSCT [79], show promising accuracies even in the ultra-lightweight range, emphasizing that a carefully designed architecture can demonstrate the same level of accuracy as a deep architecture with several times fewer parameters. Moreover, models such as BioMiFormer [115] and MBHNN [65], despite being computationally expensive, do not dominate lightweight models in terms of accuracy and sit well below the Pareto frontier, indicating that additional complexity is not reliably converting into higher performance and instead adds inference latency and training cost.

### 6.2. Challenges of MI-BCIs

Despite the significant progress made in recent years, MI-BCI classification still faces several challenges that impair performance, thus leading to low applicability. This section discusses some of those challenges.

#### 6.2.1. Limited Generalization Capability

One of the key challenges not fully addressed yet is the limited generalization of the existing models. Several models, while showcasing strong performance within specific subjects or datasets, struggle to generalize across diverse datasets or subjects. This is undesirable for practical implementation, where models need to be robust across diverse users and testing conditions.

#### 6.2.2. Subject-Independent Performance

**Figure 11:**
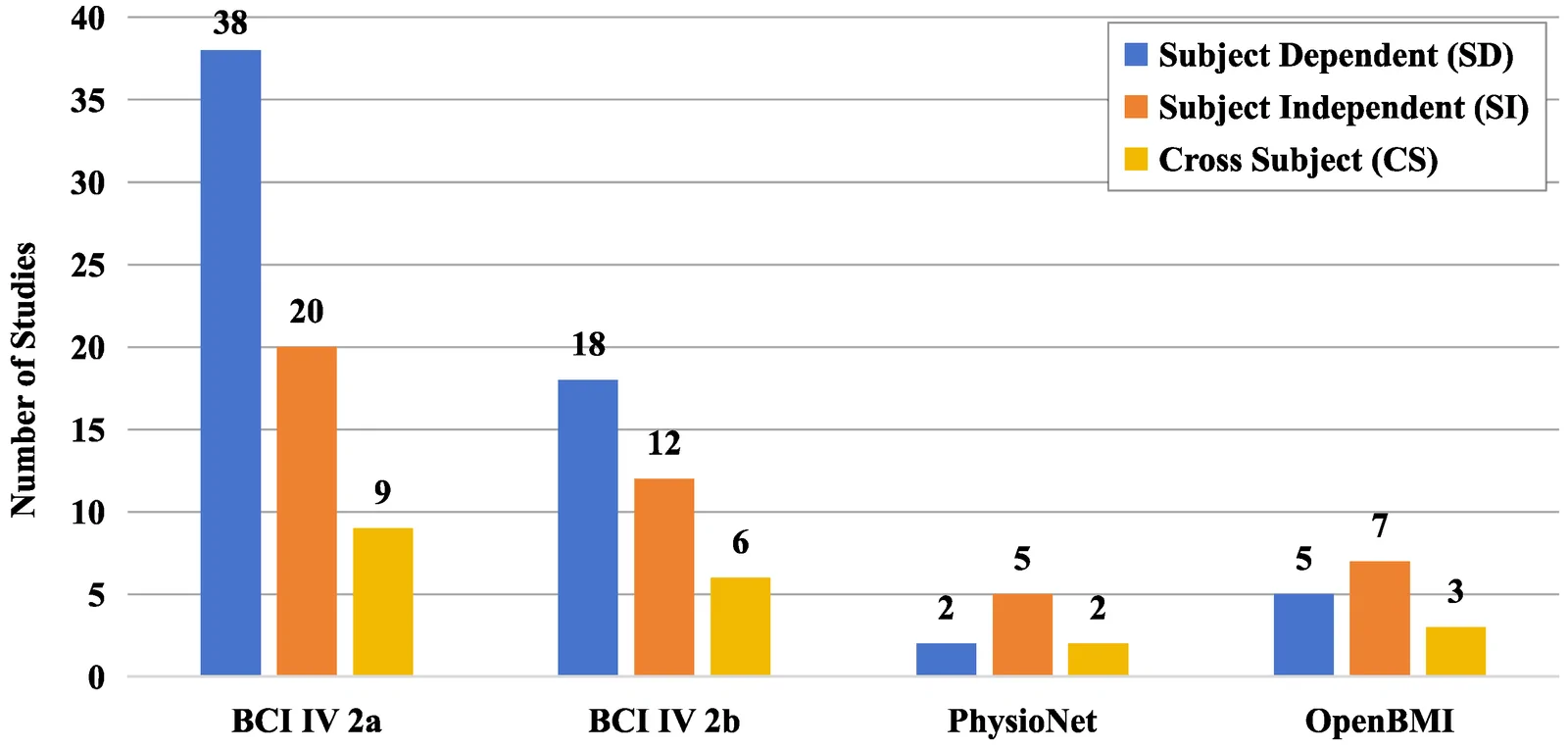
Visualization of frequency of SI, SD, and CS testing setup in the selected studies.

Another major challenge is the low subject-independent performance of MI classification models. As shown in Figure 11, most studies focus on designing subject-dependent frameworks, where feature extractors and classifiers are trained and tested on the same subject, often achieving high accuracies. However, most of these models tend to perform poorly in a subject-independent classification setup. Therefore, building subject-independent classification models is critical for designing scalable BCIs that can be used by a wider range of subjects without the need for extensive retraining and fine-tuning.

#### 6.2.3. Real-Time Application

While several MI classification models presented in the literature report impressive results, they often rely on offline datasets rather than real-time applications. Figure 5 summarizes the datasets used in this study, where BCI IV 2a is the most frequently used dataset for validating MI decoding models, followed by BCI IV 2b. Both of these datasets are offline datasets recorded in a controlled and ideal environment. Nonetheless, for BCIs to be practically useful, real-time classification is crucial, particularly in controlling devices or facilitating communication. This real-time implementation comes with additional challenges, such as the additive signal noise, computational latency, and environmental variations.

#### 6.2.4. Low Signal-to-Noise Ratio (SNR)

A persistent challenge in EEG-based MI classification is the low SNR inherent in EEG data. EEG signals are highly susceptible to various forms of artifacts and noise, including muscle activity, environmental interference, and internal brain noise, making it challenging to extract meaningful insights from the EEG data.

#### 6.2.5. Limited Data Size

Another major obstacle in MI classification research is the limited size of the available EEG datasets. Most datasets have a small size, which might lead to overfitting in DL models, where the classifier trains by learning patterns specific to the training data but fails to generalize well to new or unseen data.

#### 6.2.6. BCI Illiteracy

BCI illiteracy refers to the inability of certain users to effectively utilize BCI systems, even after training. This remains a significant hurdle to the broader adoption of MI-based BCIs, as every individual cannot generate the clear and task-specific EEG data required for training of BCI classification models.

### 6.3. Emerging Research Directions

Based on the identified challenges, the following research directions appear promising.

#### 6.3.1. Foundation Models and Self-Supervised Learning

One of the recent trends in MI classification is large-scale EEG foundation models trained through self-supervised learning on unlabeled MI-EEG data, similar to how BERT and GPT-style models were trained on large NLP data. Contrastive learning is one such strategy, where large models such as BENDR [132] and BIOT [133] learn EEG representations by discriminating signal segments. Another strategy is masked reconstruction, in which models such as EEGPT [134] and LaBraM [135] are pretrained to reconstruct masked frequency and time patches of the EEG signals. This allows the model to understand complex EEG representations from a broad corpus of cross-subject and cross-session datasets rather than learning from a limited specific dataset. These pretrained models can then be fine-tuned on task-specific, small datasets, thus directly addressing the low subject-independent performance and limited dataset size challenge addressed in Section 6.2.2 and Section 6.2.5, which not only improves robustness but also reduces the need for large labeled EEG data.

#### 6.3.2. Multimodal Models (Integration with Other Neuro-Imaging Modalities)

EEG data are susceptible to several internal and external artifacts; therefore, an area of active research is to fuse other complementary modalities, such as functional near-infrared spectroscopy (fNIRS), with EEG data to improve the spatial resolution. These fusion strategies can range from feature- and decision-level fusion to deep cross-modal attention architectures that jointly train on both EEG and fNIRS data. Reported results [136] in the literature show that exploiting two diverse streams from EEG and fNIRS data outperforms single-modality EEG classifiers on cognitive and motor tasks. Therefore, integrating EEG data with other neuro-imaging modalities to achieve better robustness requires further exploration and dedicated research.

#### 6.3.3. Synthetic Data Generation Using Diffusion Models

Diffusion models have recently emerged as powerful synthetic data generation architectures and have demonstrated higher task-level fidelity compared to GAN- and VAE-based augmentation methods [137]. These models are trained by initially corrupting a signal with additive Gaussian noise; they then generate new samples by iteratively denoising a sample drawn from pure noise to a real data distribution, thus solving the limited dataset size challenge indicated in Section 6.2.5. However, given their reported advantages compared to VAEs and GANs, their utilization in EEG data augmentation is still underexplored and could be a promising research direction.

### 6.4. Application Areas of MI-BCIs

MI-based BCIs have witnessed significant expansion in the areas in which they are applied, evolving from primarily medical applications to a wide range of non-medical domains. Initially, BCIs were primarily designed to facilitate individuals with motor disabilities in achieving better communication with external peripheral devices. However, over time, MI-based BCIs have expanded vertically, making their way into entertainment, leisure, and smart control.

#### 6.4.1. Medical Applications

BCIs have several applications in the medical field, particularly in neuro-rehabilitation for stroke patients. MI-BCIs attempt to train stroke patients’ brains to recover their lost motor function by imagining movements rather than actually performing them. Moreover, non-invasive BCIs allow individuals to practice rehabilitation in both the clinical setting and at home.

Another major application of MI-BCIs lies in the design of assistive devices that help individuals with motor disabilities maintain communication with the environment. For instance, an MI-enabled wheelchair can enable individuals with motor impairments to take left or right turns without any help using imagined movements. Moreover, MI-enabled cursor control allows users to control cursor movement through imagined movements, eventually helping them operate computers and other smart devices. Another useful application is BCI spellers that enable users to communicate using spellings of words. Moreover, BCI-enabled typing systems are helping individuals with limited mobility to write messages, thus enhancing their communication and reducing dependence on external help.

#### 6.4.2. Non-Medical Applications

In the past decade, MI-BCIs have expanded their applicability to a far wider range, with applications in virtual reality (VR), smart homes, and intelligent gaming. In VR, BCIs are being used to control virtual objects by imagined movements, thus enhancing the VR experience. Moreover, in gaming, MI-BCIs are being explored as a means to control actions or specific commands, thus enhancing the overall user experience. In smart homes, BCIs are employed to enhance the communication of users with appliances, including security systems, lights, and other devices, thus serving both individuals with disabilities and also enhancing the convenience of healthy users.

Another promising application of MI-BCIs recently explored is robotic control. MI-BCIs are helpful in controlling robots for several purposes, such as achieving industrial automation, providing remote operation or assistance, and using robots in hazardous areas. Moreover, BCI-enabled drones are being designed as they are particularly helpful in enhancing surveillance systems, search and rescue, and other drone applications. Vehicle control is also emerging as a popular BCI application, holding the potential to revolutionize the vehicle industry with MI-driven cars.

Overall, this past decade has witnessed huge growth in the MI-BCI industry and, over time, it will grow even bigger and better. This growing range of applications signifies the versatility of MI-BCIs, thus highlighting their potential to enhance the quality of life for both physically challenged and healthy individuals.

## 7. Conclusions

In conclusion, sequence, attention-based, and generative models have emerged as transformative models in the MI decoding domain, significantly enhancing the potential of BCIs in achieving a wide range of applications. This study presents a comprehensive analysis of the recently developed generative models, such as GANs and VAEs, as well as the sequence models, like RNNs, LSTMs, and transformers, emphasizing their efficacy in improving MI classification accuracy.

This review analyzes multiple studies that utilize generative and sequence models and concludes that these models have exhibited remarkable performance, surpassing traditional methods in classification performance. Overall, hybrid models, especially a combination of convolutional- and self-attention-based architectures, have demonstrated favorable performance, in terms of both accuracy as well as computational cost. It is also concluded that generative models can enhance MI classification accuracy by augmenting or reconstructing EEG data. However, despite these high levels of performance, certain challenges remain. The real-time integration of these models into functional BCI devices faces several hindrances, such as their computational complexity and the need for extensive hyperparameter tuning. Extensive adoption is also challenged by issues including inter-subject variability, the need for sufficient training data, and the low generalization performance of models among diverse environments and users.

Nonetheless, the BCI research community is actively engaged in finding solutions to address these issues. Ongoing efforts are focused on optimizing model architectures, reducing computational complexity, and developing more robust and user-friendly BCI systems. The domain of MI classification holds significant potential for a wide range of applications, particularly with the new machine learning and DL architectures. As research continues to grow, it is likely to facilitate the emergence of more accurate and accessible BCI systems, enhancing the lives of those with motor disabilities and increasing the prospects for human–computer interaction.

### Limitations

This review has certain limitations that need to be considered when interpreting its analysis. First, the literature we used was found using a few selected keywords, as mentioned in Section 2. While these keywords provide a broad coverage of the relevant literature, there is still a likelihood that a substantial share of BCI research may not have been captured, which may limit the comprehensiveness of this study. Second, our screening methodology used several filters, such as restricting our analysis to only pure MI-BCI paradigms; predefined sequence-oriented, attention-based, hybrid, and generative architectures; and studies published in the last ten years only. This screening narrowed our analysis to only 78 studies from the entire reviewed corpus, which may have limited the generalizability of the conclusions drawn from this analysis. Third, the selected studies are highly heterogeneous in their data augmentation strategies, preprocessing techniques, datasets choice, and validation protocols, which might have affected the performance comparisons across various studies, and therefore, apparent performance differences across multiple frameworks cannot always be cleanly attributed to the model architecture itself. Fourth, this analysis heavily relies on author-reported results, without including a formal assessment of the methodological quality or experimental rigor of the reviewed publications. Therefore, the performance differences between studies might be affected by variations in experimental design and reporting quality, which should be kept in mind when interpreting the findings of this study. Since no standardized assessment framework was applied to the individual studies reviewed here, the conclusions from this review should be considered as a descriptive syntheses rather than as evidence of a definitive architectures ranking. Fifth, this review may be subject to publication bias (arising from skewness of the reported performance statistics, since studies reporting weaker performance are less likely to be indexed in the searched databases), terminology-bias (studies describing conceptually relevant methods under different terminologies not taken into account), and/or duplicate-publication bias (overlapping results from the same research potentially overstating the consistency or support for any particular models). Finally, in order to take into account the recent developments in the domain, this review also contains preprints and conference paper results, which might not have undergone the same level of scrutiny as peer-reviewed publications.

## Figures and Tables

**Table 1:** Details of various BCI datasets.

Dataset Name	Trials	Channels	Subjects	Targets
BCI Competition IV 2a	288	22	9	LH, RH, BF, T
BCI Competition IV 2b	120–160	3	9	LH, RH
OpenBMI	400	62	54	LH, RH
PhysioNet	120	64	109	LH, RH, EO, BF
High Gamma Dataset (HGD)	∼1000	128	14	LH, RH, BF

**Table 2:** Non-transformer, CNN-RNN, and sequential hybrid architectures.

Ref.	Year	Model Architecture	Protocol	Methodology
[61]	2018	Cascade/Parallel CRNN: CNN + RNN	Datasets: PhysioNet and a private dataset; evaluation: CS; results: PhysioNet accuracy—98.3%, private dataset accuracy—93%.	Develops cascade and parallel convolutional–recurrent pipelines that jointly capture local spatiotemporal patterns and longer sequential dependencies from EEG representations.
[62]	2019	IncepCNN-BGRU: Inception CNN + bidirectional GRU	Dataset: BCI IV 2a; evaluation: SD; results: accuracy—76.62%; kappa—approximately 0.688.	Uses multiscale inception convolutions to extract spatiotemporal features and a bidirectional GRU to model the EEG sequence in both forward and backward directions.
[63]	2020	DWT-BiLSTM: Discrete wavelet transform + bidirectional LSTM	Dataset: BCI IV 2a; evaluation: 500 cross-validation repetitions; results: accuracy—87.14%.	Decomposes EEG into multiresolution wavelet sub-bands and feeds the resulting sequences to a bidirectional LSTM, allowing temporal information to be integrated from both directions.
[64]	2021	HDNN-TL: CNN-LSTM + transfer learning	Dataset: BCI IV 2a; evaluation: transfer learning; results: accuracy—80.0%	Learns spatial and temporal information through parallel CNN and LSTM branches, then adapts the classifier to a new subject by fine-tuning the fully connected layer with limited target data.
[65]	2022	MBHNN: Multi-branch CNN + bidirectional GRU	Datasets: BCI IV 2a and HGD; results: accuracy—86.15% and 95.04%, respectively.	Separates EEG into four MI frequency bands, learns branch-specific CNN features, and models bidirectional temporal dependencies with a BGRU supported by Seg-Swap augmentation.
[42]	2022	Transfer learning CNN-LSTM	Dataset: BCI IV 2a; evaluation: transfer learning comparison; results: accuracy—86%	Transfers pretrained CNN image backbones to EEG representations and couples their spatial features with LSTM-based temporal modeling for motor imagery classification.
[66]	2022	VAE denoising + DAE-CNN	Dataset: PhysioNet; evaluation: 10-fold cross-validation; results: accuracy—96.59%.	Uses a variational autoencoder to suppress EEG noise, a deep autoencoder to learn compact nonlinear representations, and a five-layer CNN to classify four MI tasks.
[43]	2022	CNN-LSTM feature-fusion network	Dataset: BCI IV 2a; evaluation: SD; results: accuracy—87.68%; kappa—0.8245.	Processes EEG through parallel CNN and LSTM pathways, enriches the representation with intermediate convolutional features, and fuses the spatial and temporal information before classification.
[67]	2022	SHNN: SincNet-based CNN + GRU	Datasets: BCI IV 2a and BCI IV 2b; evaluation: SI; results: accuracy—74.26% and 83.49%; kappa—0.6648 and 0.6697, respectively.	Uses CSP-windowed inputs, learnable SincNet filter banks, squeeze–excitation channel selection, CNN feature extraction, and GRU sequence modeling to support subject-independent decoding.
[68]	2023	2D CNN-LSTM	Datasets: Private EEG and BCI IV 2b; evaluation: SD; results: accuracy—93.3% and 75.4%, respectively.	Transforms segmented EEG time series into two-dimensional inputs, extracts spatial patterns with convolutional layers, and uses an LSTM to preserve the temporal ordering of successive segments.
[69]	2024	1-D MTCNN-LSTM: Multi-task CNN + LSTM	Datasets: BCI IV 2a and BCI IV 2b; results: accuracy—87.14% and 82.04%, respectively.	Introduces self-supervised auxiliary tasks within a one-dimensional multitask CNN-LSTM framework, improving the robustness of temporal EEG representations used for classification.

**Table 3:** Transformer- and Attention-Augmented Hybrid Architectures.

Ref.	Year	Model Architecture	Protocol	Methodology
[47]	2021	S3T: CNN + transformer	Datasets: BCI IV 2a and BCI IV 2b; evaluation: SD; results: accuracy—82.59% and 84.26%, respectively.	Attention-based transformation is applied across channel and temporal dimensions, with convolutional positional encoding applied to preserve local features; finally, global pooling and classification are performed.
[70]	2021	CNN + transformer + attention BiLSTM	Dataset: BCI IV 2a; evaluation: SD; results: accuracy—85.5%.	Band-specific features are extracted with a filter-bank CNN; weights informative sub-bands, and uses an attention-based bidirectional LSTM to identify discriminative temporal segments.
[71]	2022	S-CAMLP-Net: Self-supervised channel-attention MLP-Mixer	Dataset: BCI IV 2a; evaluation: SD; results: accuracy—79.43 ± 1.73%, respectively.	EEG patterns are used without labels; firstly, attention block is used to focus on important channels, and then MLP-Mixer blocks are used to combine information from different features.
[72]	2022	f-CTrans: CNN + transformer	Dataset: PhysioNet; evaluation: CS; results: accuracy—83.31% (2 classes), 74.44% (3 classes), and 64.22% (4 classes).	Combines CNN-based spatiotemporal features with positional embeddings and transformer attention to extract features before classification.
[73]	2022	Hybrid CNN-Transformer: CNN + transformer	Dataset: BCI IV 2a; evaluation: SD; results: accuracy—83.91%; kappa—0.782.	Uses convolutional layers to capture local spatial–temporal patterns and transformer self-attention to model global temporal relationships within an end-to-end motor imagery classifier.
[74]	2022	EEG Conformer: CNN + transformer	Datasets: BCI IV 2a and BCI IV 2b; evaluation: SD; results: accuracy—78.66% and 84.63%; kappa—0.7155 and 0.6926, respectively.	Compact temporal–spatial CNN is used for local EEG features and a transformer encoder for global feature extraction, with segmentation–recombination augmentation improving generalization.
[75]	2022	Multiscale convolutional transformer	Datasets: Private, BCI IV 2a; evaluation: SI; results: accuracy—62% and 70%, respectively.	Combines multiscale convolution with multi-head attention across spatial, spectral, and temporal domains, enabling a single framework to decode motor, visual, and speech imagery.
[76]	2022	ATCNet: CNN + transformer	Dataset: BCI IV 2a; evaluation: SD and SI; results: SD accuracy—85.38%; SI accuracy—70.97%.	Processes convolutional sliding windows with multi-head self-attention and temporal convolution, capturing task-relevant intervals and extended temporal dependencies in a compact network.
[77]	2023	Self-attention CNN + TFCSP	Datasets: BCI IV 2a; evaluation: SD; results: mean accuracy—79.28%	The paper introduces an architecture that combines a channel-selective self-attention CNN with time–frequency common spatial pattern features, combining learned spatiotemporal information with handcrafted representations.
[78]	2023	EGSAN: EEG graph self-attention + SVM	Dataset: BCI IV 2a; results: accuracy—86.21%; kappa—0.862 ± 0.02.	Represents EEG electrodes as a graph and applies graph self-attention to learn functional relationships between channels, after which an SVM classifies the resulting graph-level features.
[46]	2023	SMT: CNN + transformer	Datasets: BCI IV 2a, BCI IV 2b, and OpenBMI; evaluation: SI; results: accuracy—67.28%, 76.41%, and 79.76%, respectively.	Uses a lightweight CNN feature extractor and mirrored shallow transformer branches, where multi-head attention captures temporal segments for subject-independent prediction.
[79]	2023	TSCT: CNN + transformer	Datasets: BCI IV 2a and BCI IV 2b; evaluation: SD and SI; results: SD accuracy—83.3% and 89.4%; SI BCI IV 2a accuracy—69.4%.	Combines EEGNet-style temporal–spatial filtering and multibranch convolution with a transformer encoder, allowing local features to be refined using global temporal context.
[80]	2023	MI-CAT: CNN + transformer	Datasets: BCI IV 2a and BCI IV 2b; evaluation: CS; results: accuracy—76.81% and 85.26%, respectively.	Extracts shallow temporal–spatial features with CNN layers and processes source–target feature through self- and cross-attention blocks to align domain-specific representations.
[81]	2023	HSACNet: CNN + transformer	Dataset: BCI IV 2a; evaluation: SI; results: accuracy—56.75%.	Combines convolutional feature extraction with self-attention, which first learns the local features and then reweights them according to their global relevance for unseen-subject classification.
[82]	2023	ADFCNN: CNN + transformer	Datasets: BCI IV 2a, BCI IV 2b, and OpenBMI; evaluation: SD and CS; results: CS accuracy—79.39%, 87.81%, and 65.26%; SD accuracy—86.87%, 87.26%, and 84.29%, respectively.	Attention-based dual-scale fusion convolutional neural network is used. The two parallel branches are used to extract features. One extracts spatial patterns using separable convolutions and the other captures broad features using standard convolutions. Finally, after concatenation, an attention block is used to highlight important features before classification.
[45]	2023	ECA Swin Transformer: CNN + transformer	Dataset: BCI IV 2a; evaluation: SD; results: 83.91%.	Channel attention is used to prioritize informative electrodes and a Swin Transformer to model dependencies through local and shifted windows, which captures important features for classification.
[83]	2023	FB-Sinc-CSANet: Filter-bank SincNet + transformer	Datasets: BCI IV 2a, BCI IV 2b, and OpenBMI; evaluation: SI; results: accuracy—78.20%, 87.34%, and 72.03%, respectively.	Sinc convolutions are used as adaptive band-pass filters; extracts filter-bank features, and applies channel self-attention to emphasize informative EEG electrodes.
[84]	2023	st-CViT: Spatiotemporal viT	Datasets: BCI IV 2a and BCI IV 2b; evaluation: SI; results: accuracy—80.44% and 74.73%, respectively.	Processes compact convolutional EEG tokens with a Vision Transformer, providing global attention while retaining a lightweight representation for leave-one-subject-out decoding.
[85]	2023	GAT: Global adaptive transformer	Datasets: BCI IV 2a and BCI IV 2b; evaluation: CS; results: accuracy—76.58% and 84.44%; BCI IV 2b: kappa—0.6889.	Combines temporal–spatial convolutions with a global adaptive transformer, adversarial marginal alignment, and adaptive center loss to reduce subject-related distribution differences.
[86]	2023	Hierarchical transformer	Datasets: BCI IV 2a, PhysioNet, and OpenBMI; evaluation: SD and SI; results: SD/SI accuracy—90.0%/70.3%, 83.5%/80.2%, and 82.1%/81.3%, respectively.	Organizes EEG into low- and high-level transformer stages, first modeling short temporal intervals and then integrating informative features across the complete trial.
[87]	2023	Improved Wasserstein Domain-Adaptation Network: CNN + transformer	Datasets: BCI IV 2a and BCI IV 2b; evaluation: CS; results: accuracy—77.60% and 85.06%, respectively.	Combines an attention-variance feature extractor with a Wasserstein domain discriminator, aligning source and target EEG distributions for CS transfer.
[88]	2024	EISATC-Fusion: CNN + transformer	Datasets: BCI IV 2a and BCI IV 2b; evaluation: SD and CS; results: SD accuracy—84.57% and 87.58%; CS BCI IV 2a accuracy—67.42%.	The model uses an EEGNet-based inception module to extract multiscale spatial–temporal features, combined with self-attention and temporal convolution to learn important EEG patterns.
[89]	2024	MSVTNet: Multi-scale ViT	Datasets: BCI IV 2a, BCI IV 2b, and OpenBMI; evaluation: SD and SI; results: SD accuracy—82.56%, 70.30%, and 75.93%; SI accuracy—64.10%, 76.45%, and 77.41%, respectively.	Extracts multiscale convolutional tokens and applies transformers to model cross-scale and global temporal interactions, while auxiliary losses guide feature fusion.
[90]	2024	EEG-VTTCNet: ViT + CNN	Datasets: BCI IV 2a and BCI IV 2b; evaluation: SD; results: accuracy—84.58% and 90.94%, respectively.	This paper trains a Vision Transformer and temporal convolutional network, combining global attention with temporal receptive fields for motor imagery decoding.
[91]	2024	CLS: Contrastive learning + self-attention network	Datasets: OpenBMI; evaluation: SI; results: accuracy—83.70%.	Combines supervised learning with self-attention to capture temporal dependencies and learn subject-independent representations.
[92]	2024	DualDomain-AttenNet: CNN + transformer	Dataset: BCI IV 2a and BCI IV 2b; evaluation: SD and CS; results: SD accuracy—81.17% and 87.50%; CS BCI IV 2a accuracy—60.14%.	Temporal- and frequency-domain features are extracted using temporal convolutions and global filters, then applies multi-head self-attention to give attention to important features.
[93]	2024	TST-ICA: Temporal–spatial transformer + ICA	Datasets: BCI IV 2a and BCI IV 2b; evaluation: SD; results: SD accuracy—97.77% and 85.90%	Applies ICA for artifact removal and then EEG signal is passed through separate temporal and spatial transformer modules, enabling long-range dependencies to be modeled across both dimensions.
[94]	2024	Attention-based CNN with multimodal temporal fusion	Dataset: BCI IV 2a; evaluation: SD; results: BCI IV 2a accuracy—85.03%.	Different time-based features are extracted using multiple CNN layers and then given to shared attention module for important-pattern extraction.
[95]	2024	MBMANet: Multi-branch multi-attention network	Dataset: BCI IV 2a; evaluation: SD; results: accuracy—83.18%; kappa—0.776.	Processes EEG with multiple EEGNet branches, having different receptive fields and attention mechanisms, then performs multilevel concatenation to combine complementary temporal and spatial representations.
[96]	2024	Multiscale spatiotemporal self-attention network	Datasets: BCI IV 2a, BCI IV 2b, and HGD; evaluation: SD and CS; results: SD accuracy—79.20%, 85.90%, and 95.75%; CS BCI IV 2a accuracy—73.78%.	Integrates multiscale temporal convolutions with spatial and channel self-attention, emphasizing informative EEG patterns across multiple temporal resolutions.
[97]	2024	DB-ATCNet: CNN + transformer	Datasets: BCI IV 2a and PhysioNet; evaluation: SI and SD; results: BCI IV 2a SI/SD accuracy—71.34%/87.54%; PhysioNet SI accuracy—87.33% (2 classes) and 69.58% (4 classes).	Dual branch of attention block and temporal convolution is used for capturing local and global EEG patterns for subject-dependent and subject-independent decoding.
[98]	2024	TBTSCTnet: CNN + transformer	Datasets: BCI IV 2a and BCI IV 2b; evaluation: SI; results: accuracy—77.39% and 78.20%, respectively.	Combines segmentation–recombination augmentation with three temporal–spatial convolution branches and a transformer encoder, integrating multiscale local features with global contextual information.
[99]	2024	PMSA-Net: CNN + transformer	Dataset: BCI IV 2a; evaluation: SD; results: accuracy—81.40%, kappa—0.75, F1-score—0.81.	Expands EEG into the depth dimension, extracts complementary temporal–spatial features through parallel multiscale branches, and applies depth and efficient channel attention before classification.
[100]	2025	SCATNet: Sinc-CNN + CBAM + transformer encoder	Datasets: BCI IV 2a and BCI IV 2b; evaluation: SD; results: 2a accuracy—80.48% ± 10.91%, kappa—0.7397; 2b accuracy—87.08% ± 8.51%, kappa—0.7415.	Learns interpretable band-pass filters using Sinc convolution, applies grouped spatial CNNs with CBAM attention, and employs a transformer encoder to capture long-range temporal dependencies.
[101]	2025	EEG Lightformer-KD: CNN + transformer + knowledge distillation	Datasets: BCI IV 2a, BCI IV 2b, and OpenBMI; evaluation: CS; results: accuracy—78.67%, 83.24%, and 70.36%, respectively.	Distills knowledge from a frozen EEGPT teacher into a compact student model combining depthwise-separable convolution, transformer attention, and multiscale pooling through joint KL-divergence and cross-entropy loss.
[102]	2025	ECA-ATCNet: CNN + transformer + channel attention	Datasets: BCI IV 2a and PhysioNet; evaluation: SD and SI; results: BCI IV 2a SD accuracy—87.89%; PhysioNet SI accuracy—71.88%.	Parallel and efficient spatial–spectral channel attention is introduced into ATCNet, along with spike-driven ANN-to-SNN conversion, which improves feature extraction and energy-efficient deployment.
[103]	2025	CNNViT-MILF-a: CNN + ViT	Datasets: BCI IV 2a, BCI IV 2b, HGD; evaluation: SI; results (accuracy/kappa/F1): 2a 80.40%/0.739/0.804; 2b 81.11%/0.622/0.811; HGD 88.66%/0.849/0.887.	Evaluates multiple CNN-ViT fusion strategies and selects a late-fusion architecture, where ViT provides global representations while CNN extracts local EEG features, for improved spatial–temporal modeling.
[104]	2025	SMANet: SincNet + CNN + transformer + ECA	Datasets: BCI IV 2a, BCI IV 2b, and OpenBMI; evaluation: CS; results: accuracy—80.21% ± 9.66%, 84.02% ± 9.00%, and 72.70% ± 15.03%, respectively.	Learns adaptive spectral filters using SincNet, fuses multiscale spatial–temporal CNN branches, and applies efficient channel attention. Joint cross-entropy and center loss improves feature compactness.
[105]	2025	TriNet: FFT + transformer + encoder–decoder + ELM	Datasets: BCI IV 2a and BCI IV 2b; evaluation: SD and SI; results: SD accuracy—87.30% and 92.64%; SI accuracy—63.92% and 78.60%, respectively.	Builds spectral, spatial, and temporal representations through FFT, self-attention, and latent features, and concatenates them into a unified feature space, and performs classification with an ELM.
[106]	2025	EEGEncoder: CNN + transformer	Dataset: BCI IV 2a; evaluation: SD and pooled full-model evaluation; results: SD accuracy—86.46%; pooled accuracy—74.48%.	Combines modified transformer layers with temporal convolutions in parallel, capturing local EEG patterns and long-range temporal–spatial dependencies.
[107]	2025	MSCFormer: CNN + transformer	Datasets: BCI IV 2a and BCI IV 2b; evaluation: SD; results: accuracy—82.95% and 88.00%, respectively.	Applies multiscale CNN branches to extract local patterns and uses a transformer encoder to integrate these features.
[108]	2025	ConSwinFormer: CNN + transformer	Dataset: BCI IV 2a; evaluation: SD; accuracy—83.99%.	Transforms EEG signals into a 3D structure and uses a novel architecture with a CNN and Swin Transformer module to decode EEG features.
[109]	2025	DB-STFFCNet: CNN + transformer + FFT	Datasets: BCI IV 2a and BCI IV 2b; evaluation: SD; results: accuracy—83.13% and 89.54%, respectively.	A feature fusion based on a spatiotemporal feature extraction module paired with a frequency feature extraction module is used to capture fine-grained EEG features.
[110]	2025	SCGTNet: GRU + transformer	Dataset: BCI IV 2a; evaluation: SD; results: accuracy—79.47%.	A DL framework with a reweighting mechanism-based attention to extract spatial features and parallel convolutional filters to extract temporal features is designed.
[111]	2026	EEG-TriNet++: CNN + transformer + BiLSTM	Datasets: BCI IV 2a and PhysioNet; evaluation: SI and SD; results: BCI IV 2a SI/SD accuracy—72.4%/79.1%; PhysioNet SI/SD accuracy—71.3%/78.6%.	A hybrid model with three core components, channel-wise attention encoders for spatial features, BiLSTM for temporal features, and a transformer for global features, is designed to enhance EEG feature representation for efficient MI decoding.
[112]	2026	PG-MCTFormer: Temporal filter bank + transformer	Dataset: BCI IV 2a; evaluation: SD; results: accuracy—85.08%.	A unified framework consisting of a rhythm-aware temporal filtering and a dual-scale spatial modeling is proposed for reliable MI decoding.
[58]	2026	CCST: CNN + transformer	Datasets: BCI IV 2a, BCI IV 2b, and PhysioNet; evaluation: SI; results: accuracy—68.27%, 76.61%, and 71.70%, respectively.	This paper combines compact convolutional feature extraction with hierarchical Swin Transformer blocks and then uses windowed attention to model local electrode interactions and long-range temporal dependencies.
[113]	2026	HATNet: CNN + transformer	Datasets: BCI IV 2a, BCI IV 2b, OpenBMI, and HGD; evaluation: SD and SI; results: SD accuracy—81.25%, 86.65%, 69.57%, and 96.20%; SI accuracy—60.88%, 80.79%, 76.28%, and 73.95%, respectively.	This paper integrates CNN spatiotemporal features with channel attention, entropy pooling, and a dynamic transformer, while cross-layer residual fusion preserves information across network depths.
[114]	2026	BCI-Transformer: CNN + transformer	Datasets: BCI IV 2a, BCI IV 2b, and OpenBMI; evaluation: SI; results: accuracy—77.24%, 88.42%, and 79.64%, respectively.	Convolutional stem is used for rhythmic and topographic features, followed by localized spatiotemporal transformer attention and manifold alignment for calibration-free decoding.
[115]	2026	BioMIFormer: CNN + transformer + BiGRU	Datasets: BCI IV 2a and BCI IV 2b; evaluation: SD, cross-session; results: accuracy—81.06% and 83.82%, respectively.	BiGRU, transformer, and convolutional are used in parallel for temporal, spatial, and spatiotemporal modeling, then the features are passed through transformer attention and adaptive weighting.
[116]	2026	TFCA-TransNet: CNN + transformer + channel attention	Datasets: BCI IV 2a, BCI IV 2b, and HGD; evaluation: cross-session; results: accuracy—84.38%, 89.39%, and 95.85%, respectively.	The model combines temporal signals, FFT amplitude–phase information, and depthwise-separable spatial features, and then channel attention is performed before giving the data to a transformer.
[117]	2026	CTER: CNN + transformer + ridge ELM	Datasets: BCI IV 2a, BCI IV 2b, and OpenBMI; evaluation: SD; results: accuracy—84.90%, 86.77%, and 79.26%, respectively.	Parallel CNN and spatial transformer branches are used to learn local features, then their outputs are concatenated for classification by an RSA-optimized ridge extreme learning machine.
[118]	2026	CT-MIFNet: CNN + transformer	Datasets: BCI IV 2a and BCI IV 2b; evaluation: hold-out and 5-fold cross-validation; results: hold-out accuracy—81.67% and 86.75%; 5-fold cross-validation—83.48%.	Generates temporal, frequency, and spatial views using FFT and multiscale convolutions, then introduces cross-covariance attention to fuse information efficiently across the three views.
[119]	2026	MRTSANet: CNN + transformer + BiLSTM	Dataset: BCI IV 2a; evaluation: SD and SI; results: SD accuracy—80.52% ± 9.19%; SI accuracy—62.20% ± 12.12%.	Partitions EEG channels into anatomically defined regions, extracts multiscale features using independent CNN branches, and models temporal dependencies with BiLSTM followed by temporal attention.
[120]	2026	MSHANet: STFE + talking-head self-attention + DSEA + TCN	Datasets: BCI IV 2a and BCI IV 2b; evaluation: SD and SI; results: SD accuracy—83.56% ± 9.44%, 89.75% ± 8.35%; SI—69.93% ± 11.04%, 81.85% ± 5.35%	Combines multiscale spatiotemporal feature extraction with talking-head self-attention, dynamic squeeze-and-excitation attention, and temporal convolution. A spatial structure encoder preserves electrode topology information.

**Table 5:** Reported computational-efficiency metrics for MI-EEG studies on BCI IV 2a dataset.

Ref.	Model	Parameters	FLOPs/MACs	Inference Time	Training Time	Accuracy
[58]	CCST	150.96 K	38 M	2.41 ms	-	SI: 68.27
[113]	HATNet	29.04 K	24.98 M	3.84 ms	-	SD: 81.25
[114]	BCI-Transformer	820.00 K	14,600.00 M	3.80±0.20 ms per trial	-	CS: 77.24
[115]	BioMIFormer	2070.00 K	100.00 M	36.12 ms per sample	-	SD: 81.06
[105]	TriNet	18.00 K	-	-	-	SD: 87.30
[107]	MSCFormer	145.90 K	-	-	-	SD: 82.95
[102]	ECA-ATCNet	440K	-	-	-	SD: 87.89
[46]	SMT	29.84 K	-	5.08 ms	-	SI: 67.28
[77]	Self-Attention CNN + TFCSP	-	-	110.53 ms/trial	-	SD: 79.28
[79]	TSCT	13.40 K	-	-	-	SD: 83.30
[92]	DualDomain-AttenNet	-	-	1740.00±50.00 ms	-	SD: 81.17
[65]	MBHNN	521.09 K	-	-	16.32 min/subject	SD: 86.15
[47]	S3T	8.68 K	-	-	-	SD: 82.59
[45]	ECA Swin Transformer	-	-	17.04 min/subject	-	SD: 83.91
[71]	S-CAMLP-Net	226.62 K	1.00 M	-	-	SD: 79.43
[83]	FB-Sinc-CSANet	27.40 K	-	-	14.03 h (whole dataset)	SI: 78.20
[97]	DB-ATCNet	150.00 K	-	-	-	SD: 87.54
[74]	EEG Conformer	-	-	-	0.27 s/epoch	SD: 78.66
[88]	EISATC-Fusion	26.37 K	-	-	-	SD: 84.57
[84]	st-CViT	136.00 K	-	-	-	SI: 80.44
[85]	GAT	8.20 K	23.10 M	-	-	CS: 76.58
[64]	HDNN-TL	-	-	-	16 s/500 iterations	SD: 80.00
[75]	MS-TSformer-DS	65.11 K	-	-	-	SI: 70.00
[76]	ATCNet	115.20 K	-	-	-	SD: 85.38
[67]	SHNN	-	-	2030.00 ms/trial	6.66 min/subject	SD: 74.26
[101]	EEG Lightformer-KD	127.00 K	-	-	-	CS: 78.67
[120]	MSHANet	28.48 K	21.32 M	3.24 ms	-	SD: 83.56
[104]	SMANet	-	-	-	190.35 min	CS: 80.21
[112]	PG-MCTFormer	128.9 K	211.29 M	4.29 ms	-	SD: 85.08
[109]	DB-STFFCNet	29.5 K	66.33 M	0.479 ms	0.61 s/per epoch	SD: 83.13
[110]	SCGT	55.4 K	1.12 M	-	-	SD: 79.47
[111]	EEG-TriNet++	1870 K	126 M	23.5 ms	-	SI: 72.4

## Data Availability

No new data were created or analyzed in this study. Data sharing is not applicable to this article.

## Abbreviations

The following abbreviations are used in this manuscript:

BCI	Brain–computer interface
CNN	Convolutional neural network
CSP	Common spatial pattern
DL	Deep learning
EEG	Electroencephalography
GAN	Generative adversarial network
GRU	Gated recurrent unit
LSTM	Long short-term memory
MI	Motor imagery
RNN	Recurrent neural network
SNR	Signal-to-noise ratio
SSVEP	Steady-state visually evoked potential
VAE	Variational autoencoder
SI	Subject independent
EO	Eyes open
SD	Subject dependent
EC	Eyes closed
CS	Cross-subject
BCI IV 2a	BCI Competition IV dataset 2a
BCI IV 2b	BCI Competition IV dataset 2b
T	Tongue
HGD	High Gamma Dataset
LH	Left hand
RH	Right hand
BF	Both feet
